# Maps of variability in cell lineage trees

**DOI:** 10.1371/journal.pcbi.1006745

**Published:** 2019-02-12

**Authors:** Damien G. Hicks, Terence P. Speed, Mohammed Yassin, Sarah M. Russell

**Affiliations:** 1 Centre for Micro-Photonics, Department of Physics and Astronomy, Swinburne University of Technology, Hawthorn, Victoria 3122, Australia; 2 Bioinformatics Division, Walter & Eliza Hall Institute of Medical Research, Parkville, Victoria 3052, Australia; 3 Peter MacCallum Cancer Centre, Parkville, Victoria 3052, Australia; 4 Department of Pathology and Sir Peter MacCallum Department of Oncology, University of Melbourne, Parkville, Victoria 3050, Australia; University of Michigan, UNITED STATES

## Abstract

New approaches to lineage tracking have allowed the study of differentiation in multicellular organisms over many generations of cells. Understanding the phenotypic variability observed in these lineage trees requires new statistical methods. Whereas an invariant cell lineage, such as that for the nematode *Caenorhabditis elegans*, can be described by a lineage map, defined as the pattern of phenotypes overlaid onto the binary tree, a traditional lineage map is static and does not describe the variability inherent in the cell lineages of higher organisms. Here, we introduce lineage variability maps which describe the pattern of second-order variation in lineage trees. These maps can be undirected graphs of the partial correlations between every lineal position, or directed graphs showing the dynamics of bifurcated patterns in each subtree. We show how to infer these graphical models for lineages of any depth from sample sizes of only a few pedigrees. This required developing the generalized spectral analysis for a binary tree, the natural framework for describing tree-structured variation. When tested on pedigrees from *C. elegans* expressing a marker for pharyngeal differentiation potential, the variability maps recover essential features of the known lineage map. When applied to highly-variable pedigrees monitoring cell size in T lymphocytes, the maps show that most of the phenotype is set by the founder naive T cell. Lineage variability maps thus elevate the concept of the lineage map to the population level, addressing questions about the potency and dynamics of cell lineages and providing a way to quantify the progressive restriction of cell fate with increasing depth in the tree.

## Author summary

Multicellular organisms develop from a single fertilized egg by sequential cell divisions. The progeny from these divisions adopt different traits that are transmitted and modified through many generations. By tracking how cell traits change with each successive cell division throughout the family, or lineage, tree, it has been possible to understand where and how these modifications are controlled at the single-cell level. This helps address questions about, for example, the developmental origin of tissues, the sources of differentiation in immune cells, or the relationship between primary tumors and metastases. Such lineages often show large variability, with apparently similar founder cells giving rise to different patterns of descendants. In addition, questions about the range of accessible cell types at different stages of the lineage tree are actually questions about lineage variability. To characterize this variation, and thus understand the lineage at the population level, we introduce lineage variability maps. Using data from worm and mammalian cell lineages we show how these maps provide quantifiable answers to questions about any developing lineage, such as the potency of progenitor cells and the restriction of cell fate at different stages of the tree.

## Introduction

The cells of developing organisms differentiate into their specialized types by integrating signals from their present surroundings with instructions inherited from their ancestors. This interplay of mechanisms generates the pattern of phenotypes that emerges in the cell lineage tree [1]. Measuring this phenotypic pattern requires recording two types of information: the phenotype of each cell and the family relationships between the cells. The result is called a lineage map [2]. Lineage maps illustrate the successive bifurcations in phenotypes that underpin a particular differentiation pathway, providing an invaluable guide for experiments investigating the mechanisms involved in fate determination [3]. Development in the nematode *Caenorhabditis elegans* is the classic example of how the lineage map can help untangle the roles of pre-programmed instruction and cell-to-cell communication [4–6] in cellular differentiation.

A crucial use of the lineage map is to identify the common ancestors of cells sharing a particular fate. This indicates how deeply within the lineage that fate was specified. Successfully locating common ancestors depends first on being able to identify the subclones associated with a phenotype. If a phenotype is clonal, meaning exclusive to a single subclone, we can associate that phenotype with a single bifurcation at the most recent common ancestor; if it is non-clonal, bifurcations at multiple ancestors were likely involved (note that even for simple organisms like *C. elegans*, most phenotypes are non-clonal—see Table 1). Much of the logic for interpreting lineage maps and inferring differentiation pathways can be automated [7, 8]. However, these techniques are difficult to implement in the presence of phenotypic variability.

**Table 1:** Characteristics of some cell lineage patterns. Organisms are listed in order of increasing complexity. Lineages are characterized in terms of whether cell fate is exclusive to a subclone, the degree of phenotypic variability, and whether the lineage tree measurement is ordered. Lineages from higher organisms are generally unordered, have high variability, and may or may not be clonal.

*Species*	*Cell origin (tissue)*	*Clonal*	*Variability*	*Ordered*	*Ref*.
Worm	Embryonic (germ)	✔	low	✔	[5]
	Embryonic (pharynx)	✘	low	✔	[5]
Leech	Embryonic (epidermis)	✘	low	✔	[11]
Zebrafish	Embryonic (various)	✘	high	✘	[19]
Mouse	Embryonic (various)	✘	high	✘	[20]
	Lymphoma	✔	high	✘	[21]
	B-lymphocyte	✘	high	✘	[14]

### Variability in cell lineages

The lineage map is a concept born from the study of invariant lineages, such as that for *C. elegans*, where the fixed pattern of phenotypes can be found, in principle, by tracking the family tree, or pedigree, from a single founder cell. When pedigrees are highly variable, however, seemingly identical founder cells can give rise to different patterns of descendants. Which of these represents the lineage map? Simply averaging the phenotype at each lineal position by pooling across multiple pedigrees may not give a representative pattern since the averaging will suppress correlations between lineal positions. Furthermore, the variability between pedigrees, which reflects the potency of founder cells, is an important quantity itself and cannot be represented in a lineage map. While lineage variability is minimal in simple organisms such as *C. elegans* [9, 10] and leech [11], it is greater in higher organisms such as insects and vertebrates [1, 12] and is significant in mammalian cells of clinical importance such as stem cells [13] and lymphocytes [14, 15]. Such increased variability likely plays a role in being able to respond effectively to environmental changes. Given the additional variation inherent in molecular-level measurements [16] it is becoming increasingly important to extend the concept of the lineage map to account for variability.

A fundamental property of any lineage tree measurement, which is crucial when studying variability, is whether it is ordered or unordered. We define a lineage tree to be *ordered* if the labels used to distinguish the lineal positions of two daughter cells (sisters) are meaningful. This gives each daughter a unique identity. The tree is *unordered* if these labels are arbitrary, making the daughter positions unidentifiable. This distinction has significant consequences in a statistical analysis. For example, in an unordered tree consisting of a mother *A* and its arbitrarily-labeled daughters *B* and *C*, we should not distinguish between the properties of positions *B* and *C* when comparing different pedigrees; nor should we distinguish between the mother-daughter relationships *A-B* and *A-C*.

The *C. elegans* lineage is an example of an ordered lineage. Here each daughter is labeled by its orientation, at the time of division, with respect to the developing organism [4, 5, 17]. Daughters thus have meaningful labels. For example, a mother labeled ‘Epl’ divides into ‘Epla’, the anterior daughter, and ‘Eplp’ the posterior daughter. In higher organisms, it can be difficult or impossible to find a meaningful way to label each daughter in a pair. For example, in the *in vitro* murine T-lymphocyte pedigrees discussed in this paper, the orientation of the mouse, even if it were known, can hardly be expected to be a useful way to distinguish between daughter cells.

Now, if the phenotype pattern is invariant, a single complete pedigree measurement represents the lineage map and being unordered (or not) does not matter. However, when comparing variable pedigrees, the unidentifiability of daughter positions can significantly affect the ability to detect phenotypic patterns. For example, simply averaging different pedigrees to enhance subtle bifurcation patterns risks instead canceling these patterns if the pedigrees are not ordered the same way [18].

Since the majority of pedigree measurements from higher organisms are both variable and unordered [1] (see Table 1), an important question is whether the concept of a lineage map is even useful anymore. How can we associate fate specification with fixed lineal positions when the pattern of descendants varies from one apparently identical founder to the next? Clearly a statistical approach is required.

### Previous statistical approaches

A number of statistical methods have been developed to analyze variable, unordered lineage trees. Though these approaches do not directly address the question of how to construct a lineage map, many of them address aspects of the problem.

The bifurcating autoregressive model [22, 23] was developed to estimate mother-daughter and daughter-daughter correlations using a sample of unordered pedigrees from either *E. coli* or tumor cultures. The model was later used to analyze data from ordered pedigrees to test for lineage asymmetry [24, 25]. This stationary, parametric model allowed for daughters to be conditionally dependent (with respect to their common mother) but forced cousins and more distant relatives to be conditionally independent (with respect to their most recent common ancestor). The subsequent discovery that cousins could be conditionally dependent motivated a theory of cellular inheritance involving deterministic, nonlinear dynamics in lymphoblasts [26]. However, such distant intragenerational dependence might instead be interpreted as a delay between fate specification and expression, where a phenotype that has been specified in a mother and its daughters is not expressed until its four granddaughters. These analyses illustrate the importance of having lineages that are large enough, and a model that is general enough, to examine correlations of distant relatives [27]. They also remind us that simple branching process models, which we here define to be those assuming conditional independence of daughters, do not properly represent the correlations in a lineage, a fact that was established in early lineage analyses [28, 29]. Although population numbers can be satisfactorily modeled using branching processes [30], allowing for sibling correlations can affect the inferred population dynamics [14, 31].

As we indicated earlier, identifying a subtree, or subclone, of shared phenotypes is the first step to inferring where fate is specified. This idea forms the basis of methods to study cell state transitions in bacterial cells or mouse embryonic stem cells [32, 33], where phenotypic similarity among relatives in the same generation was used to infer how much earlier in the pedigree a transition occurred. A similar idea was used in hematopoietic stem cells to infer the multi-generational delay between when an invisible molecular decision occurred and when its effect was expressed as a surface marker [34]. These models assume that cell states transition over timescales that are slow compared to the cell cycle duration; alternatively they could be synchronized to cell divisions [35]. Note that, in a lineage map, the generation of a cell is a meaningful quantity, representing the number of divisions since the founder cell, whether that be a zygote, a naive lymphocyte, or some progenitor initiated with a particular stimulus. Thus any model of a developing lineage must be non-stationary.

Several other approaches to statistical lineage analysis have been reported recently. A factor graph method was used to model conditional dependence between daughters [36], with the goal of testing whether pre-programmed instruction or differential cell death was responsible for differentiation of hematopoietic progenitor cells; direct inference of Nanog expression, a pluripotency factor, was used to understand its dynamics in embryonic stem cell lineages [37]; and, a parametric characterization of lineage patterns has been applied to achieve early identification of hematopoietic stem cells [38]. However, these methods are of less relevance to our question of how to build a statistical lineage map.

### Outline

Major efforts are underway to improve the throughput and quality of lineage measurements (see reviews [13, 39–42] and commentary [43–45]). Recent breakthroughs have resulted in a wealth of data from automated microscopy-based [19, 46–48] and sequencing-based [20, 49–57] techniques. While the technological barriers for these measurements are severe, there are significant barriers to the analysis of the data as well. As we have discussed, there is currently no way to construct a useful lineage map from variable, unordered pedigrees. Yet, according to Shapiro [40], “Central unresolved problems in human biology and medicine are in fact questions about the human cell lineage tree: its structure, dynamics, and variability during development, growth, renewal, aging and disease.” It is important then to find a way to generalize the concept of the lineage map to the population level.

In this paper we propose a solution by introducing lineage variability maps. These involve the variances of, and covariances between, every position in the tree. A key idea is that, to interpret lineage patterns, it is not only the phenotypic values at each lineal position that are important, but also the phenotypic associations between different lineal positions. By developing a generalized spectral analysis for binary trees, we show how to estimate variability maps for a lineage of any depth using measurements from only a few, unordered pedigrees. For complete data, our approach is a non-parametric one, involving first and second moments of the data but assuming no distribution function. We could thus, alternatively, refer to these maps as second order lineage maps.

The rest of the paper is organized as follows. In the Methods section we describe essential aspects of the data used in this paper, the analysis framework and labeling convention, and how to estimate all pairwise associations by employing constraints on symmetry and sparsity. In the Results section we interpret the covariance matrix in terms of graphical models, called lineage variability maps, and show how these can be used to understand fate restriction and expression throughout the lineage. In the Discussion section we examine the implications and future prospects of this analysis.

## Methods

### Ethics statement

All experiments using mice were performed in accordance with the Animal Experimentation Ethics Committee of the Peter MacCallum Cancer Centre (Approval E427) and mice were sacrificed by anaesthetic overdose.

### Lineage data

Data from 3 types of lineages are analyzed in this paper. Experimental data from T cells provide an example of a lineage with significant variability and no obvious structure. Previously-published data from *C. elegans* are the example of a lineage with complicated but highly-reproducible structure. Finally, a simulated, stationary branching process provides the benchmark of a featureless, variable lineage and to test the accuracy of the inference procedure. In more detail:

**T cells** New lineage measurement on CD8^+^ T cells from GFP:OT-1 transgenic mice. Naive cells, expressing a T cell receptor for SIINFEKL peptide from ovalbumin, interact with peptide-pulsed bone marrow-derived dendritic cells to activate clonal expansion [58]. Cells and their descendants are tracked using time-lapse fluorescence microscopy and analyzed using custom software [59]. Although multiple phenotypic traits were recorded, in this paper the only trait analyzed is the average area of a dividing cell over its lifetime. Note that only dividing cells were used in the analysis; cells whose fate is unknown, or which died, were counted as missing data. For the early generations used in this study, the numbers of cell deaths were negligible so there was no need to account for cell death explicitly. 19 replicate families were used.
**Worm** Published [60] embryonic lineage data from the RW10425 transgenic strain of *C. elegans*. In this strain the *PHA-4* gene for pharyngeal and intestinal tissue is tagged with green fluorescent protein whose lifetime-averaged expression intensity is used as the phenotypic trait in our analysis. Gut differentiation occurs early during embryogenesis, with *PHA-4* expression beginning by generations 7 and 8. There are 10 replicate pedigrees. Note that, although these lineage data are ordered, we treat them as unordered in order to compare results with the other unordered datasets in this study.
**Branching Process** Simulated lineages from a stationary branching process. 20 replicate pedigrees are used, with a missing data fraction of 20% assumed. Here we define a branching process to be one where the correlation between mothers and daughters is *h* and daughters are conditionally independent with respect to their common mother. Then, the correlation between any two lineal positions *ς* and *ς*′ is *h*^*D*(*ς*,*ς*′)^, where *D*(*ς*, *ς*′) is the lineage distance between them. For example, the correlation between sisters is *h*^2^ and between cousins is *h*^4^ and so on. In this study we assume *h* = 0.8. As will be shown in Section “Lineage variability maps”, the underlying graphical model of partial correlations for this branching process is a binary tree. This is generally not the case for real lineages.

**Fig 1:**
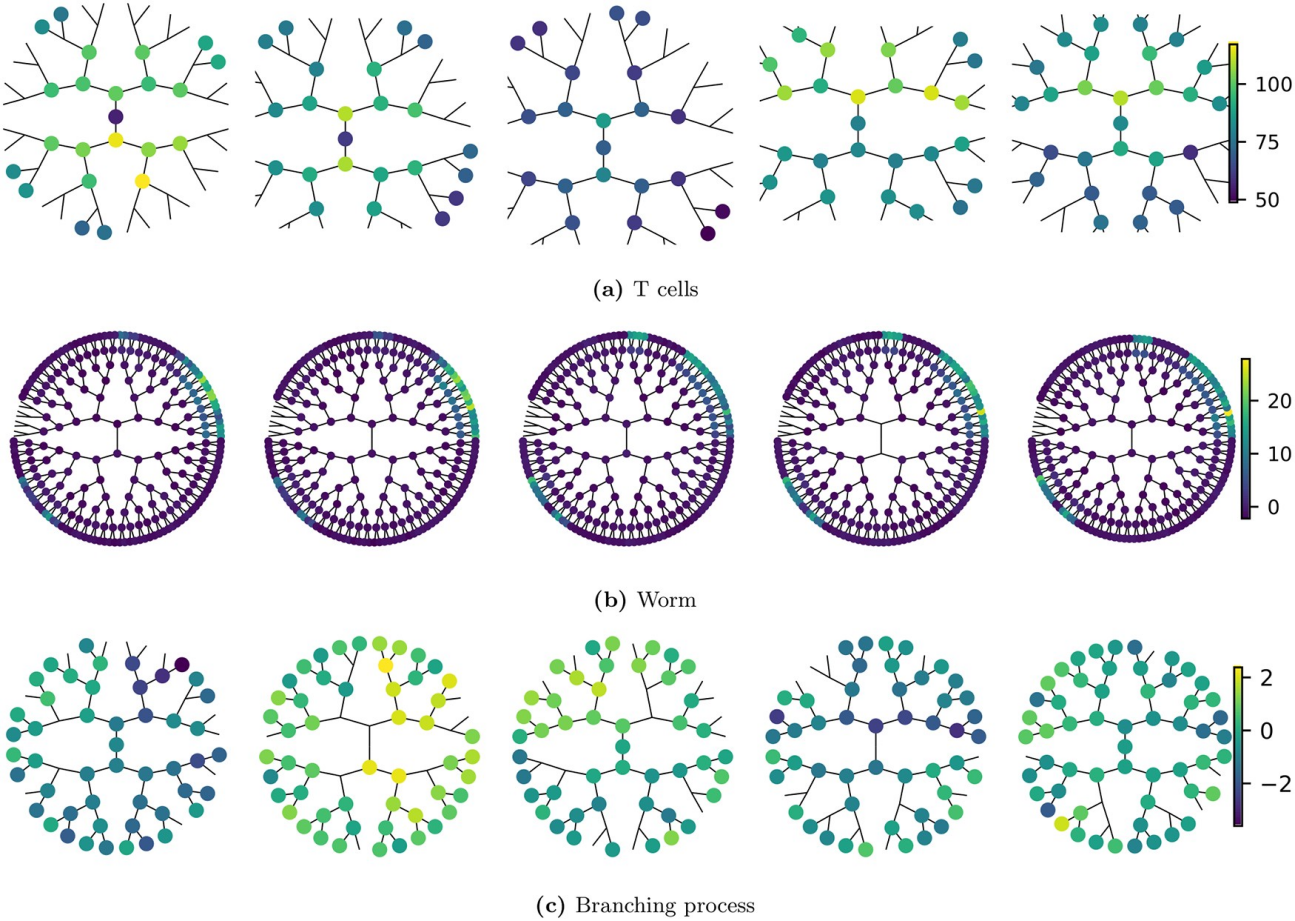
Illustration of pedigree data. Shown are 5 sample pedigrees from each of the 3 lineage types. Each pedigree originates from a different founder cell and is, for compactness, drawn as a radial tree. Each node on the tree represents a cell, where node color reflects the strength of the cell phenotype under analysis (liftetime-averaged cell size for T cells, *PHA-4* expression intensity for *C. elegans*). For T cells the root node is the naive cell while for the worm lineage the root node is the zygote (labelled P0). The absence of a node on the tree represents a missing data point. Labels for each worm cell position are given in Section S1.6 in S1 Appendix. Data shown here, and throughout the paper, are provided in the supporting information. Note how the worm pedigrees display clear, invariant patterns whereas the T-cell pedigrees (and the branching process) have no obvious repeatable structure.

**Fig 2:**
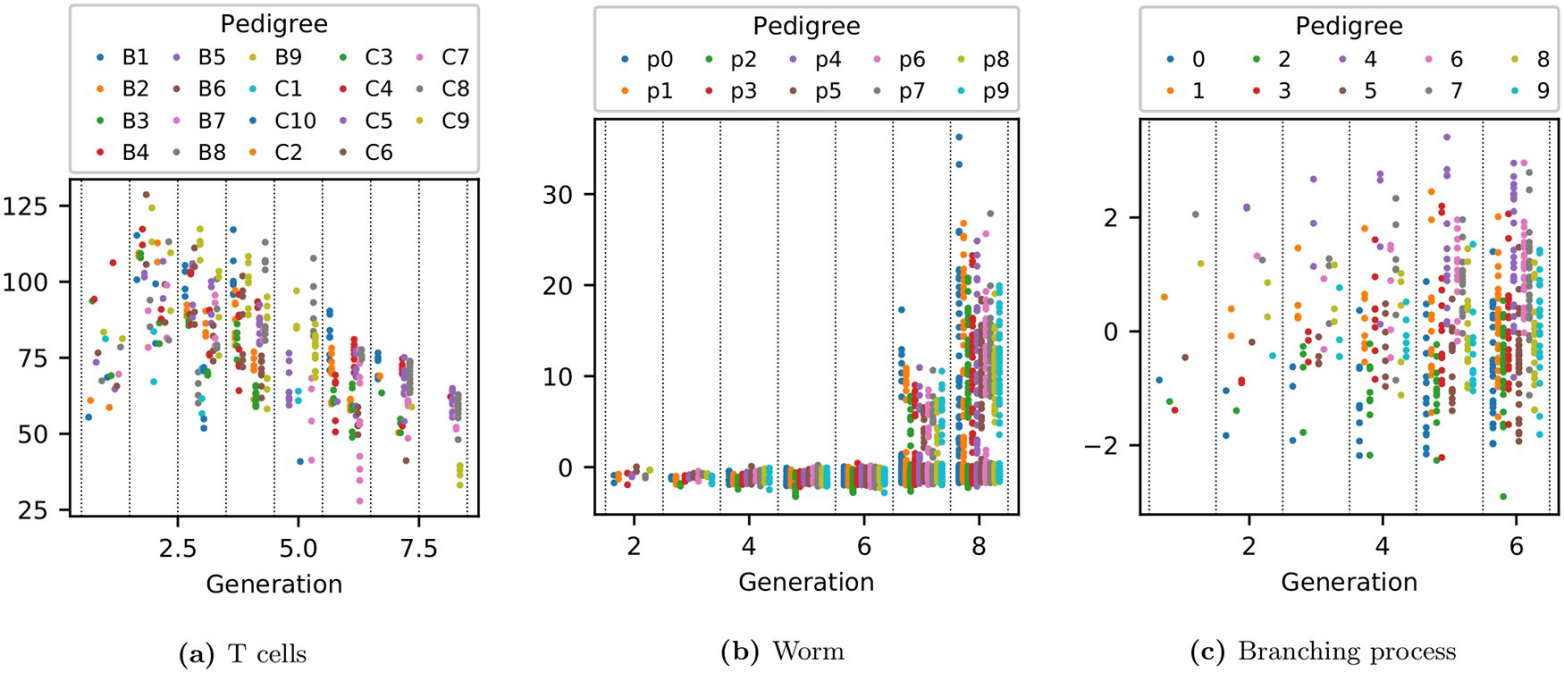
Expression of each phenotype as a function of generation. The vertical axis represents the strength of expression for each measured phenotype. For T cells this is the lifetime-averaged cell area in *μ*m^2^; for *C*. *elegans* it is the lifetime-averaged intensity of green fluorescent protein used to tag *PHA-4* expression.

Sample pedigrees from these 3 lineage types are shown in Fig 1 while the expression of each phenotype as a function of generation is shown in Fig 2.

### Analysis framework and labeling conventions

In this study, lineage data are regarded as repeated measurements on pedigrees arising from individual founder cells, each selected at random from a population of similar cells. We restrict our attention to modeling a single trait from pedigrees subject to the same conditions. A sample consisting of multiple replicate pedigrees can then be represented by a two-factor array (*Y*_*ij*_), where *i* has *n* levels corresponding to the number of pedigrees and *j* has *p* levels corresponding to the number of lineal positions within a pedigree. With no meaningful distinctions among pedigrees (they are all of the same cell type and subject to the same conditions) we assume they are independent and identically-distributed replicates. The data can thus be represented by a matrix ***Y*** with *n* replicates (rows) and *p* variables (columns).

**Fig 3:**
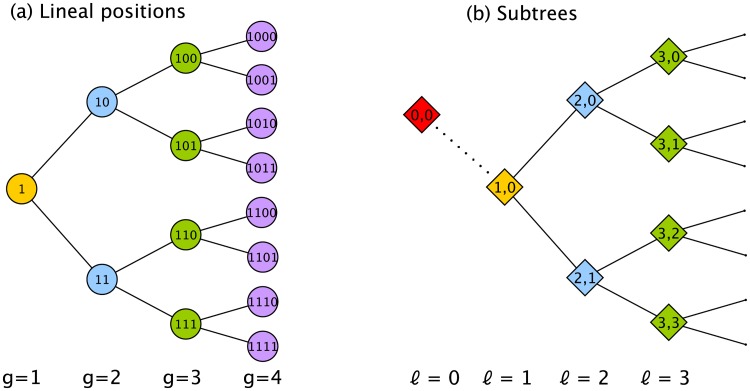
Labeling convention for a lineage tree. (a) Each lineal position is labeled with a binary number. The founder of the tree is located at generation *g* = 1. (b) Each subtree is labeled with two indices (*ℓ*, *τ*) representing the longitudinal (*ℓ*) and transverse (*τ*) coordinates of its root. Because, as we discuss later, roots of subtrees are associated with sources of variation we need to create a ‘subtree’ located outside the lineage, called (0, 0) (in red), to represent variation among pedigrees. Note that, in an unordered tree, *τ* values are unidentifiable and will often be ignored.

Each of the *p* dimensions corresponds to a lineal position. We use a binary number to label each position so that, for example, the first 3 generations are labeled as founder (1), daughters (10, 11), and granddaughters (100, 101, 110, 111), where each label thus encodes the lineal position. We will also need to define a nomenclature for generations and subtrees. Generations, *g*, refer to the depth in the tree where the founder cell is defined to be at generation *g* = 1. Subtrees are defined by two indices, (*ℓ*, *τ*), where *ℓ* refers to the longitudinal coordinate and *τ* to the transverse coordinate of the root node (see Fig 3). In our convention, the subtree at *ℓ* = 1 is the entire tree. As we will show, subtrees will be associated with sources of variation, and we need to define a ‘subtree’ at *ℓ* = 0 that sits outside the lineage and represents variation among lineages. This concept does not exist for a lineage map but is essential for a lineage variability map since different pedigrees may not be the same.

Often in lineage measurements there are many more lineal positions (*p*) than there are pedigrees (*n*). Thus *p* ≳ *n*, with the disparity getting exponentially worse with the number of generations studied. Performing reliable inference when *p*/*n* > 1 is an open research question [61]. Best results are achieved when prior knowledge of the problem can be incorporated.

In the next section we describe increasingly more sophisticated steps to reduce the effective dimensionality of the inference calculation, first by exploiting known symmetry properties and then by using observed sparsity properties. Our goal is to identify a scheme where the number of replicates required is independent of the number of generations studied. This will allow variability maps over many generations to be constructed using data from only a few pedigrees.

### Covariance estimation

The essential idea for this analysis is to measure second-order variation throughout the lineage by estimating the variance of, and covariance between, every lineal position. This population covariance matrix **Σ** for the lineage involves no assumption about the underlying distribution. It involves just the first and second order moments of the data.

#### Unstructured covariance

Let ***y*** be a *p*-dimensional random variable representing the single trait for each lineal position. A naive method for estimating the covariance matrix for ***y*** is to assume it has no structure. This means that only data from the same lineal position in different pedigrees can be pooled. The sample mean ($\bar{y}$) and (biased) sample covariance (***S***) are given by

$$\begin{array}{c} \bar{y}=\frac{1}{n}\sum_{i=1}^{n}Y_{i},\;S=\frac{1}{n}\sum_{i=1}^{n}Y_{i}Y_{i}^{\prime }-\bar{y}\;{\bar{y}}^{\prime },\;\;Y_{i}\in R^{p},i=1,\cdots ,n, \end{array}$$ (1)

where ***Y***_*i*_ is the data vector from pedigree *i*. This results in the usual estimates of the population mean, ***μ***, and population covariance matrix, $\hat{\Sigma }$,

$$\begin{array}{c} \hat{\mu }=\bar{y},\;\hat{\Sigma }=S. \end{array}$$ (2)

This is not a practical way to estimate **Σ** since, as is well known, ***S*** will not be positive definite unless *n* > *p*. To appreciate why this is a prohibitive limitation for lineage data, we examine the complexity of the problem using 3 measures: the effective number of dimensions *p*_eff_, the number of unknown variance-covariance parameters $N_{\Sigma }$, and the minimum number of replicates *n*_min_ required to ensure $\hat{\Sigma }$ is positive definite. These are given by

$$\begin{array}{cc} p_{\text{eff}} & =p,\;N_{\Sigma }=p\left(p+1\right)/2,\;n_{\text{min}}=p+1. \end{array}$$ (3)

Now because the number of lineal positions for a complete tree of *G* generations grows exponentially as *p* = 2^*G*^ − 1, this means that the number of dimensions, the number of unknowns, and, most importantly, the number of replicates required, *n*_min_, increases exponentially with the number of generations being studied. This makes the unstructured covariance matrix impractical for analyzing trees. As we invoke more constraints, we will examine the reduction in these complexity measures. For example, although *p*_eff_ = *p* for this unstructured case, with symmetry structure *p*_eff_ < *p*.

For the analysis to be practical, *n*_min_ should be small and independent of *G*. Then **Σ** can be estimated up to any generation *G* with a modest number of pedigrees *n*_min_. To achieve this, our approach is to identify constraints associated with symmetry and sparsity that are specific to the problem of tree-structured variation.

#### Symmetry

To understand how symmetry invariance constrains tree-structured variation, we start with intuitive arguments for why certain covariance matrix elements must be equal in an unordered tree. This gives rise to a particular structured form for **Σ**. We then describe how the framework of symmetry invariance formalizes this intuition and reveals the independent (orthogonal) components underlying this structured form. The result is a nonparametric spectral analysis for trees that facilitates both inference and interpretation in tree-structured data.

#### Structured covariance matrix

To reduce the number of unknowns in **Σ**, we begin by identifying a pattern of shared means, variances, and covariances that arise in the unordered tree. This allows pooling of data within a pedigree, in addition to the pooling between pedigrees already used in the unstructured covariance estimate.

For the case of first moments, the pattern of shared elements is found by recognizing that, for an unordered tree, lineal positions within the same generation are unidentifiable. Equivalently, we could say that the labels identifying members of the same generation are not meaningful. Thus all members within a generation must be assigned the same mean. For example, the mean vector for a 3-generation tree is

$$\begin{array}{cccccccc} & 1 & 10 & 11 & 100 & 101 & 110 & 111\\ \mu_{G}= & (q_{1} & q_{2} & q_{2} & q_{3} & q_{3} & q_{3} & q_{3})\prime \end{array},$$ (4)

where the subscript G denotes a quantity with symmetry structure, *q*_*g*_ is the mean of a cell in generation *g*, and we have explicitly written the cell labels above each element. It is self-evident that data can be pooled within generations to improve the estimate of these shared means.

Note how, because the tree is unordered, the only information in the first moment of the data is the average of each generation. Other details about the lineage pattern have been lost. *Thus, in unordered trees, we must look at second moments of the data if we want to understand lineage patterns at the population level*.

For the case of second moments, the pattern of shared elements is found by recognizing which relationships are equivalent. For example, as we mentioned in the introduction, the two mother-daughter relationships between generation 1 and 2 must be assigned the same covariance since there is no way to distinguish between the two. We can generalize this intuition by adopting a labeling scheme that incorporates the generation of each cell in a pair and the generation of their Most Recent Common Ancestor (MRCA). For example, the pair of cells 10 and 110, which have 1 as their MRCA, should be identified with the 3-index ‘231’, where the first two indices specify the generation of each cell (2 and 3) and the third index specifies the generation of their MRCA (1). Now since the 3-index for a different cell pair 11 and 101 is also ‘231’, the two covariances must be equal.

Note how our 3-index scheme identifies the specific generations of both cells and their MRCA, not just the lineage distance between the two cells. This is necessary because, for non-stationary variation in a tree, specific generations are meaningful, not just generational differences. For example, we need to allow for the possibility that sisters in generation 3 have a different statistical association than do sisters in generation 2, even though the lineage distance (between sisters) is the same.

Applying this labeling scheme to each variance and covariance element, the following structured covariance matrix emerges for a 3-generation tree

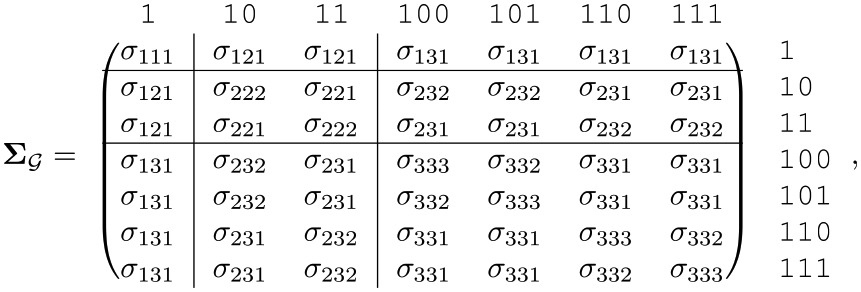
(5)
where the subscript G denotes a covariance matrix with shared elements. Improved covariance estimation can thus be achieved by pooling across matrix elements which have the same 3-index.

Note that the outer product of the structured mean, $\mu_{G}\mu_{G}^{\prime }$, has a pattern of shared elements that are bounded by the lines in Eq 5. The shared parameters in this less complex pattern are identified by the first two indices of the 3-index in Eq 5. This highlights how $\Sigma_{G}$ describes the structure of variation that is *in addition to* that due to generational trends seen in Fig 2.

We emphasize that assuming shared variances and covariances is necessary because, in an unordered tree, we have no information to assume otherwise. *We are certainly not assuming that the biology of the lineage tree is symmetric*. When we assume shared parameters for an unordered tree we are following the same reasoning as when we assume random effects, rather than fixed effects, for batched data when the labels for different batches are not meaningful (see e.g. p.21 [62]).

#### Permutation invariance

**Fig 4:**
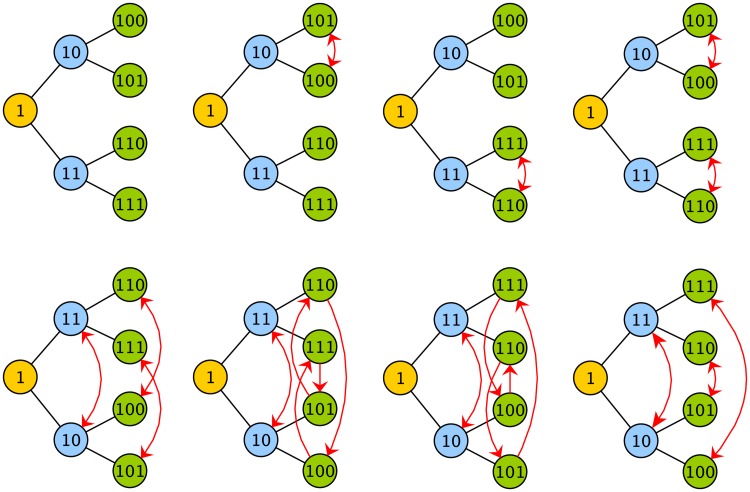
Permutation symmetry of a tree. Here the 8 allowed permutations of a tree with 3 generations are shown. These permutations, which involve the swapping of labels starting from the original arrangement in the top left corner, are allowed because they do not change the relationships in the tree. For example, consider the swapping of labels between sisters 101 and 100 (second from left in top row). Despite the swap, those lineal positions still have the same sister and mother. After any of the 8 permutations shown here, every lineal position still has the same mother, sister, cousins, granddaughters etc. In other words, the lineage tree relationships are invariant to this set of permutations.

This pattern of shared means, variances and covariances can be found more formally from symmetry considerations. An object is defined to have a symmetry if it remains invariant under the actions of a group (see Weyl [63] for the classic introduction). A lineage tree has a symmetry because (the action of) permuting daughter subtrees does not change the relationships between any of the lineal positions. After the permutation, every cell still has the same mother, daughters, cousins and so on (see Fig 4). The permutation has changed no essential information about the tree.

For our purposes, the key idea is that **Σ** remains invariant under such permutations of subtrees (since the symmetry group of **Σ** is a subgroup of the symmetry group of ***μμ***′ we can focus our attention on the symmetry group of **Σ**). Quantifying this intuitive idea involves group representation theory, where matrix multiplications are used to represent symmetry operations [64]. For example, if ***D***_*s*_ is the (*p*-dimensional) permutation matrix representing the action *s* of the group G, then the permutation *s* of the variables in ***y*** is represented by ***D***_*s*_ ***y***. The same permutation of variables in the matrix **Σ** is represented by $D_{s}\Sigma D_{s}^{\prime }$, where such conjugation by ***D***_*s*_ is necessary to permute both rows and columns.

The condition that **Σ** be invariant under the action of any member of G can thus be stated as

$$\begin{array}{c} D_{s}\Sigma D_{s}^{\prime }=\Sigma ,\;\forall s\in G. \end{array}$$ (6)

Any symmetry-invariant (i.e. G-invariant) **Σ** thus belongs to the set

$$\begin{array}{c} W_{G}=\left\{M\in R^{p\times p}|\;D_{s}MD_{s}^{\prime }=M\;\forall s\in G\right\}, \end{array}$$ (7)

referred to as the fixed point subspace of the group G [65]. This is the set of all matrices that are invariant with respect to the group.

#### Group-averaged covariance

A standard technique for transforming an unconstrained matrix **Σ** into one that is symmetry invariant is the group-average or Reynolds operator (see p. 74 [66]) given by

$$\begin{array}{cc} P_{G}\left(\Sigma \right) & =\frac{1}{\left|G\right|}\sum_{s\in G}D_{s}\Sigma D_{s}^{\prime },\;P_{G}:R^{p\times p}\to W_{G},\\ & =\Sigma_{G}, \end{array}$$ (8)

where |G| is the order of the group (the number of group elements). This projects the matrix onto the fixed point subspace by averaging over shared elements (referred to as the orbits) of **Σ**. It is straightforward to check that the pattern that arises from $P_{G}\left(\Sigma \right)$, when G is the symmetry group of the tree, is the same as that shown in Eq 5. Thus, averaging **Σ** over all its allowed permutations (members of the group) generates a structured covariance matrix that is invariant to (any further) permutations of the group.

Although the group-average (Eq 8) automatically generates the correct structured covariance for the symmetry group, it is not a practical method for tree-structured data since the number of permutations, |G|, grows super-exponentially with *G* [67]. The method is thus more of a conceptual bridge, connecting the symmetry formalism to the covariance structure, than a practical method for deriving the covariance structure itself.

To convert from unstructured to structured means, variances and covariances it is more practical to pool elements by following the shared index structure derived previously (Eqs 4 and 5). Nevertheless, we will still refer to this action of pooling across shared elements as the operation P(), however it is accomplished in practice. Thus, to find the structured sample mean and covariance,

$${\bar{y}}_{G}=P(\bar{y}),$$ (9)

$$S_{G}=P(S).$$ (10)

#### Generalized spectral analysis

The true benefit of the symmetry formalism is in how it can reduce the original high-dimensional problem into independent lower-dimensional problems that have scientific meaning (see p.161 [68]). This is achieved through a linear transformation from the set of original variables to the set of natural variables defined by the symmetry of the system. The most well-known example of this is the spectral decomposition of stationary time series data where the underlying symmetry is cyclic and the corresponding natural variables are the Fourier components. Decomposition of a system into its natural variables is thus called generalized spectral analysis, or simply spectral (or harmonic) analysis [68] and has been used in many areas of science and engineering [64].

Formal application of generalized spectral analysis to covariance estimation has been discussed recently [65, 69]. To motivate its application to a complete tree, here we briefly summarize two well-known types of spectral decomposition, Fourier analysis and the analysis of variance (ANOVA), showing how the underlying symmetry of the system defines a linear transformation that diagonalizes the structured covariance matrix.

**Fig 5:**
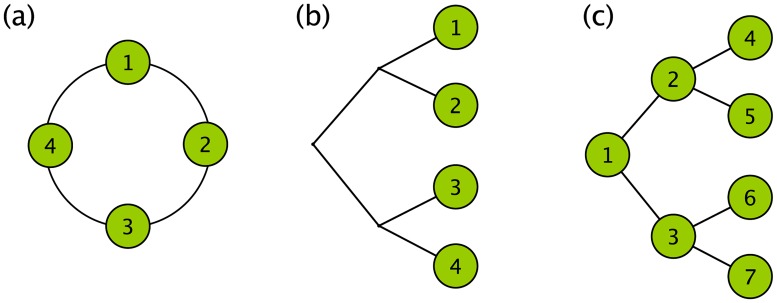
Cyclic and tree-structured symmetries. (a) A cyclic symmetry structure is one that remains invariant under a shift of all the variables (around the circle in the figure shown) that preserves their relative ordering. This cyclic symmetry defines the discrete Fourier transform. (b) A tree symmetry structure is one that remains invariant under permutations within groups and permutations of groups. This symmetry gives rise to ANOVA for nested pairs and also defines the Haar wavelet transform. It is applicable when it is just the leaf nodes that are of interest. (c) When all the nodes of a tree are of interest, the underlying symmetry is still that for the tree. The associated transformation is derived in this paper and discussed in the next section.

*Fourier analysis*. Consider the 4-variable system with the cyclic symmetry shown in Fig 5a. These variables could be a temporal sequence where the absolute value of time is not meaningful. Any cyclic shifting of the variables, which does not change in their relative ordering, does not change the system. The covariance matrix then has a circulant structure

$$\begin{array}{cc} \Sigma_{G}^{F} & =[\begin{array}{cccc} a & b & c & b\\ b & a & b & c\\ c & b & a & b\\ b & c & b & a \end{array}]. \end{array}$$ (11)

It is well-known that the circulant structure defines a unitary transformation matrix called the discrete Fourier transform (DFT) matrix which, for 4 variables, is given by

$$\begin{array}{c} F=\frac{1}{2}[\begin{array}{cccc} 1 & 1 & 1 & 1\\ 1 & -i & -1 & i\\ 1 & -1 & 1 & -1\\ 1 & i & -1 & -i \end{array}]. \end{array}$$ (12)

Each column represents a natural variable for the cyclic symmetry group, better known as a Fourier basis vector. Using ***F*** to transform $\Sigma_{G}^{F}$ into this natural basis results in a diagonal matrix

$$\begin{array}{c} \Sigma_{\Omega }^{F}=F^{†}\Sigma_{G}^{F}F=[\begin{array}{cccc} a+2b+c & \cdot & \cdot & \cdot \\ \cdot & a-c & \cdot & \cdot \\ \cdot & \cdot & a-2b+c & \cdot \\ \cdot & \cdot & \cdot & a-c \end{array}]. \end{array}$$ (13)

called the spectral covariance, where the diagonal elements represent the spectrum. Thus, the circulant-structured matrix is transformed into the spectral covariance using the DFT matrix.

*ANOVA on nested pairs (Haar wavelet analysis)*. Now consider a system consisting of nested batches of variables, a standard problem in the analysis of variance, or variance components analysis. Consider the case of 2 batches each containing 2 variables. This structure can be depicted as the leaves on a binary tree shown in Fig 5b. The symmetry operations for this structure are the permutations within groups and permutations of groups, or, as we discussed earlier, the exchange of daughter subtrees. The covariance matrix invariant under these symmetry operations has the form

$$\begin{array}{cc} \Sigma_{G}^{H} & =[\begin{array}{cccc} a & b & c & c\\ b & a & c & c\\ c & c & a & b\\ c & c & b & a \end{array}], \end{array}$$ (14)

which was given in the bottom right corner of Eq 5. The matrix that diagonalizes this structure,

$$\begin{array}{c} H=\frac{1}{2}[\begin{array}{cccc} 1 & 1 & \sqrt{2} & 0\\ 1 & 1 & -\sqrt{2} & 0\\ 1 & -1 & 0 & \sqrt{2}\\ 1 & -1 & 0 & -\sqrt{2} \end{array}], \end{array}$$ (15)

is known as the Haar (wavelet) transform matrix. Each column defines a natural variable for the tree symmetry and represents a source of variation or a wavelet component. Using ***H*** to transform $\Sigma_{G}^{H}$ into this natural basis results in a (diagonalized) spectral covariance

$$\begin{array}{cc} \Sigma_{\Omega }^{H} & =H^{†}\Sigma_{G}^{H}H=[\begin{array}{cccc} a+b+2c & \cdot & \cdot & \cdot \\ \cdot & a+b-2c & \cdot & \cdot \\ \cdot & \cdot & a-b & \cdot \\ \cdot & \cdot & \cdot & a-b \end{array}], \end{array}$$ (16)

where the diagonal elements are known as the components of variance (if we regard this from the ANOVA perspective), or the Haar wavelet spectrum (if we regard this as wavelet analysis). Here there are 3 sources of variation: between trees (*a* + *b* + 2*c*), within trees (*a* + *b* − 2*c*), and within subtrees (*a* − *b*).

We emphasize that the change-of-basis matrices ***F*** and ***H*** are defined by the symmetry of each system. They transform the original variables into a set of non-interacting natural variables (Fourier or Haar wavelet components) which define the meaningful components of variance. It was Tukey [70] who first showed that Fourier decomposition can be regarded as a branch of variance components analysis (see Speed [71] for more extensive discussion).

It is worth pointing out how this diagonalization, or eigendecomposition, of the covariance matrix, relates to traditional principal components analysis. In generalized spectral analysis, the eigenvectors (given by the columns in ***F*** and ***H***), or, more precisely, the eigenspaces, are determined by the *structure* of **Σ** and do not depend on its entries. In addition, the eigenvalues are linear functions of the entries. Neither of these properties are true, in general, for principal components analysis.

#### Generalized spectral analysis of a complete tree

Having examined the case of a tree where only the leaf nodes are of interest (Fig 5b), we now examine the case where all positions in the tree are of interest (Fig 5c). For the complete tree, we already know the structured covariance $\Sigma_{G}$ (see Eq 5). Our tasks then are to derive the change-of-basis matrix, interpret the natural variables, and calculate the spectral covariance.

Formal derivation of the change-of-basis matrix, ***T***, for a complete tree is shown in Section S1.2 in S1 Appendix. This represents the generalization of the Haar transform matrix ***H*** to a complete tree. For a 3-generation tree it is given by

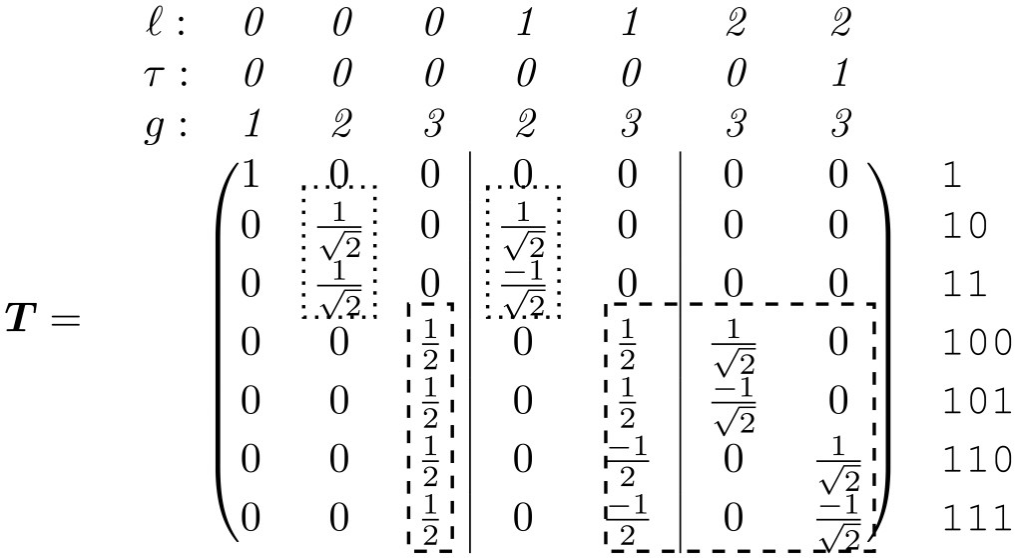
(17)
where the columns, as usual, define the natural variables.

There are two equivalent ways to interpret these natural variables: from the ANOVA perspective, and from the wavelet perspective. From the nested ANOVA perspective, each natural variable is associated with a source of variation (*ℓ*, *τ*) located at the root of a subtree. From the wavelet perspective, *ℓ* and *τ* correspond to the dilation and translation indices, respectively, for the wavelet coefficients (see e.g. [72]). In both perspectives, because we are considering a tree with multiple generations, we need a third index, *g*, specifying the generation in which the variation is observed in order to uniquely identify each natural variable (see Fig 3 for the labeling convention). The 3-index label (*ℓ*, *τ*, *g*) is given above each column in Eq 17, with vertical lines used to partition the different *ℓ*.

Extending the change-of-basis matrix to 4 generations gives

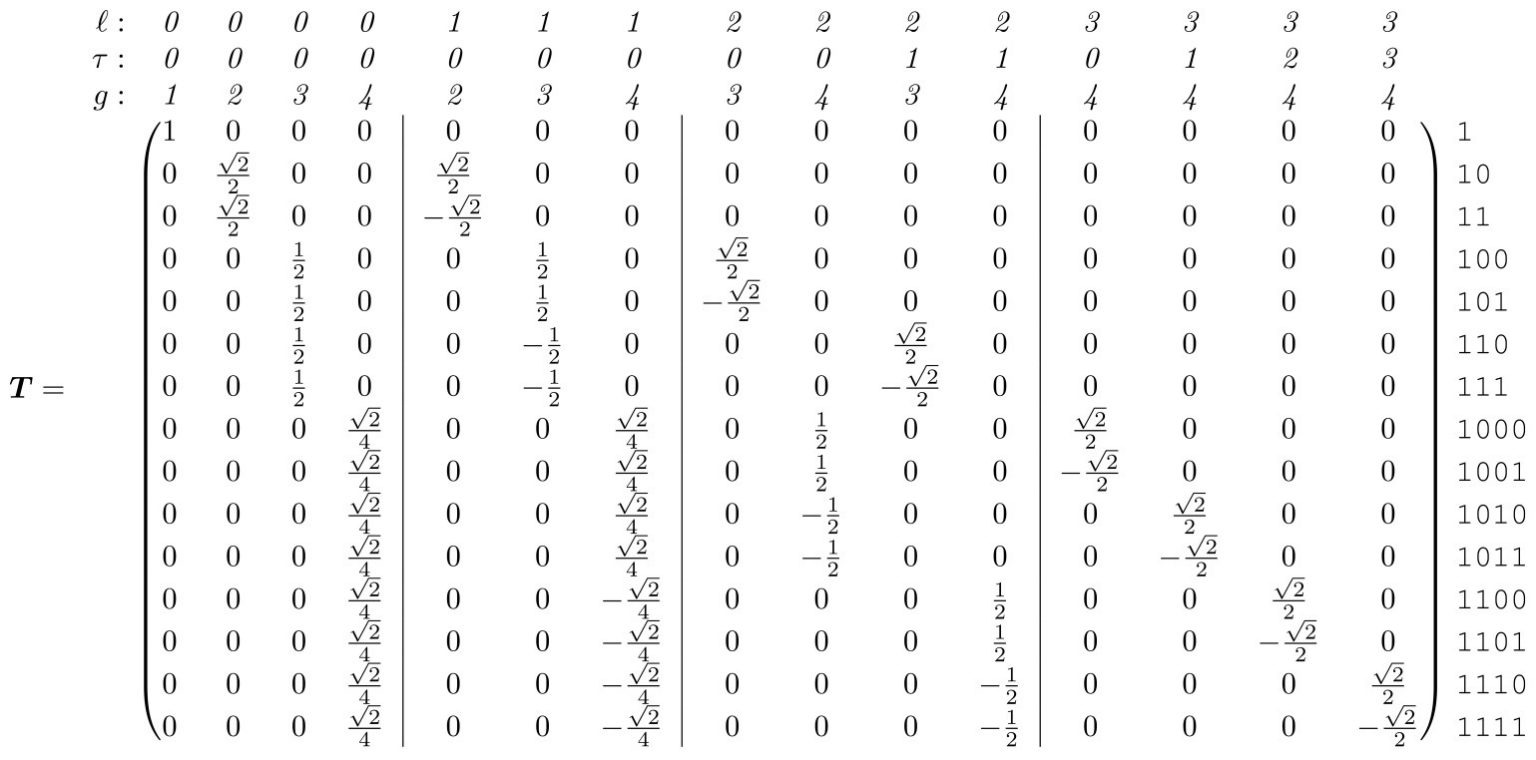


**Fig 6:**
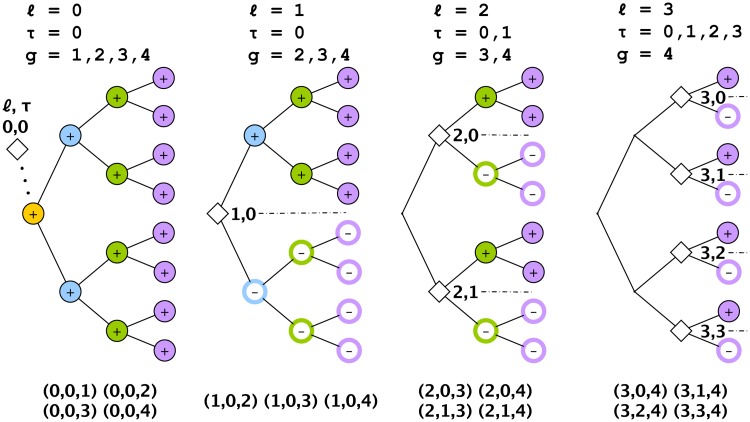
Construction of the natural variables for a tree with 4 generations. Each natural variable is identified by a source of variation (*ℓ*, *τ*), corresponding to the root of a subtree, and a generation *g*. The + and − at each lineal position illustrate how the original variables are combined to form a natural variable. The 15 natural variables thus defined by the 3-tuple (*ℓ*, *τ*, *g*) are listed in the bottom row. Since the *τ* coordinates are indistinguishable, only 10 of the natural variables (those with *τ* = 0, say) are unique.

**Fig 7:**
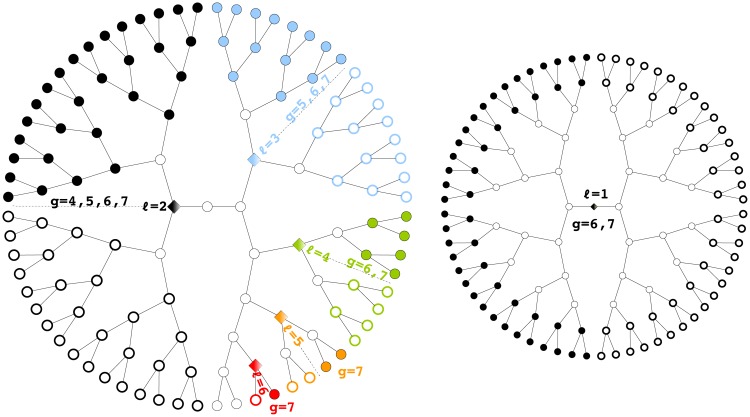
Bifurcated subtrees. Patterns on a tree can be described in terms of natural variables, or elemental components, examples of which are shown here. Each component is a bifurcated pattern centered on a subtree (*ℓ*, *τ*) and expressed in a generation *g* (where *τ* is ignored in an unordered tree). For example, the blue/non-blue bifurcated pattern occupies a subtree rooted at *ℓ* = 3 and observed at generations 5, 6, and 7. Note that *ℓ* = 1 variation (on the right) is a bifurcation across the whole pedigree. Variation among different pedigrees would be labeled with *ℓ* = 0.

A visual representation of how these natural variables are constructed from the original variables is shown in Fig 6 for the case of a 4-generation tree. This emphasizes how the *ℓ*-coordinate of the source of variation characterizes the scale of the pattern. Fig 7 shows a few examples of the natural variables to illustrate how they are convenient, elemental components for describing tree-structured variation.

The natural variables thus correspond to patterns of bifurcated expression on subtrees, or, more succinctly, *bifurcated subtrees*. These are the analogs to Fourier components, and could in fact be referred to as generalized Fourier components for the tree. Thus it is not subtrees that are the fundamental units of expression in a binary tree but rather bifurcated subtrees.

The natural variables are not particularly surprising: they are just those one would define in a nested ANOVA or Haar wavelet analysis if each generation were considered separately. Perhaps more surprising is their arrangement in ***T***: although Eq 17 contains every column of the Haar transform matrix for generations 2 (dotted lines) and 3 (dashed lines), these matrices are not incorporated simply as a direct sum. Instead, representation theory demands that we group the natural variables by (*ℓ*, *τ*). When we do this and apply ***T*** to $\Sigma_{G}$ from Eq 5 we get a *block*-diagonalized spectral covariance,

ΣΩ=T†ΣGT=(ξ11(0)ξ12(0)ξ13(0)ξ12(0)ξ22(0)ξ23(0)ξ13(0)ξ23(0)ξ33(0)..................ξ22(1)ξ23(1)ξ23(1)ξ33(1)..............ξ33(2)..ξ33(2)), (18)

where each block corresponds to a source of variation *ℓ* and its associated generations *g*, where *g* > *ℓ*. Here we label matrix elements as $\xi_{gg^{\prime }}^{\left(\ell \right)}$ where subscripts refer to the pair of interacting generations, *g* and *g*′ (there is no need to use *τ* as a label since elements differing only in *τ* have identical values). Note how the components of variation that we encountered on the diagonal in $\Sigma_{\Omega }^{H}$ (Eq 16), where only third generation variables were of interest, are here labeled as $\xi_{33}^{\left(0\right)}$, $\xi_{33}^{\left(1\right)}$, and $\xi_{33}^{\left(2\right)}$. They are still on the diagonal but are grouped with their counterparts from generations 1 and 2.

**Fig 8:**
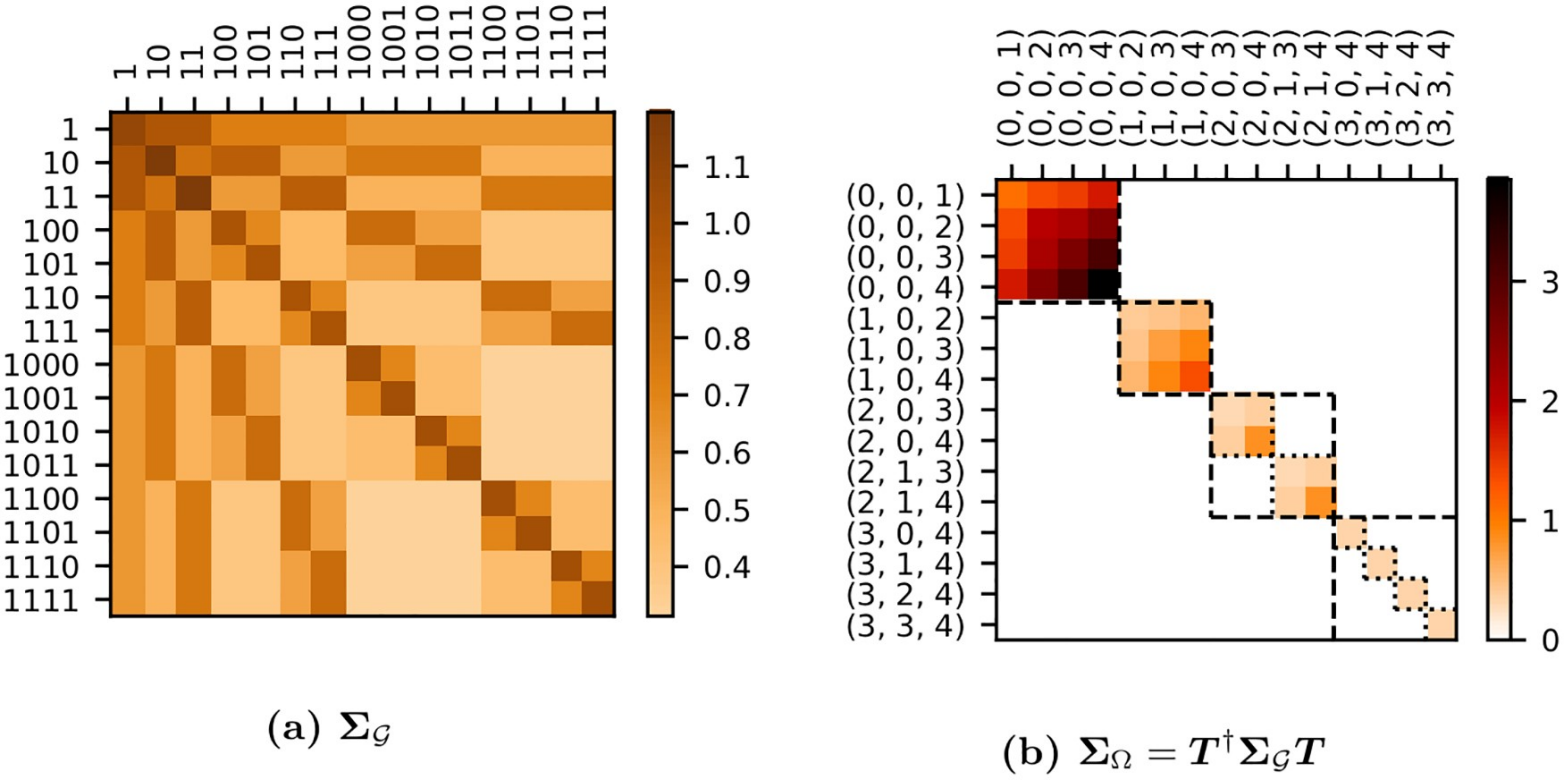
Heat maps of $\Sigma_{G}$ and Σ_Ω_ for a complete tree. This example was taken from the first 4 generations of the branching process. Natural variables along the axes of **Σ**_Ω_ are given in the format (*ℓ*, *τ*, *g*). Isotypic blocks are bounded by dashed squares and correspond to a given *ℓ*. Irreducible blocks correspond to a source of variation (*ℓ*, *τ*) and are bounded by a dotted square. For *ℓ* = 0 and 1 the isotypic and irreducible blocks coincide since there is only one *τ* index value.

To better appreciate the block-diagonal structure of **Σ**_Ω_, we show it as a heat map for the case of a 4-generation tree (Fig 8b) along with the corresponding $\Sigma_{G}$ (Fig 8a). This emphasizes how each block *ℓ* is further block-diagonalized by *τ*. In the terminology of group representation theory, *ℓ* identifies an isotypic subspace while *τ* identifies an irreducible subspace—a subset of the isotypic subspace. In Fig 8b, the isotypic blocks are bounded by dashed lines, while the irreducible blocks are bounded by dotted lines.

The primary benefit of identifying the spectral transformation for the complete tree is that **Σ**_Ω_ contains all the information in $\Sigma_{G}$ but in a much simpler form. Having pooled data to estimate ${\hat{\Sigma }}_{G}$ one simply performs the linear transformation to get ${\hat{\Sigma }}_{\Omega }$.

We pause briefly to examine how this generalized spectral analysis for a complete tree is analogous to traditional Fourier analysis for a time series. As we mentioned, bifurcated subtrees are the natural variables for a binary tree and are thus analogous to sine and cosine waves. Any pattern on a tree, whether or not it is clonal, can thus be defined as a superposition of bifurcated subtrees. This idea is useful when trying to interpret non-clonal lineage patterns: whereas a clonal pattern is associated with a single subtree, a non-clonal pattern is a superposition of multiple subtrees.

Another analogy is between the ordering of the tree and the phase of a time series. Our ability to average different trees regardless of their ordering is similar to the ability to average the spectra of different time series each having unknown (and potentially different) starting phases. One knows that to pool data across different time series, one should average their spectra, not the different time series themselves. Other analogies are shown in Table 2.

**Table 2:** Generalized spectral analysis. Well-known quantities in Fourier analysis have their direct analogs in the spectral analysis of a tree.

*Fourier analysis*	*Tree analysis*
Sine, Cosine waves	Bifurcated subtrees
Phase	Ordering of the tree
Auto-covariance	Structured covariance, $\Sigma_{G}$ (Eq 1)
Discrete Fourier Transform matrix	Change-of-basis matrix, ***T*** (Eq 17)
Power spectrum	Spectral covariance, **Σ**_Ω_ (Eq 18)

#### Complexity of the structured covariance

Spectral decomposition shows that the high-dimensional covariance estimation problem involving shared parameters in ${\hat{\Sigma }}_{G}$ is equivalent to several, lower-dimensional covariance estimation problems given by the irreducible blocks in ${\hat{\Sigma }}_{\Omega }$. We can use this to calculate the complexity of ${\hat{\Sigma }}_{\Omega }$ as we did for the unstructured covariance (Eq 3).

Because each unique irreducible block is an independent, unstructured estimate of a covariance matrix, the effective number of dimensions, *p*_eff_, is given by summing the number of dimensions for each *unique* irreducible subspace. The number of free parameters in the covariance matrix, $N_{\Sigma }$, is found by summing the number of parameters in each *unique* irreducible block. The minimum number of replicates required, *n*_min_, is found from the dimensionality of the largest irreducible block (*ℓ* = 0). Thus

$$p_{\text{eff}}=\sum_{\ell =0}^{G-1}\left(G-\ell \right)=\frac{G(G+1)}{2}=O\left(G^{2}\right),$$ (19)

$$N_{\Sigma }=\frac{1}{2}\sum_{\ell =0}^{G-1}\left(G-\ell \right)\left(G-\ell +1\right)=\frac{G}{6}\left(G+1\right)\left(G+2\right)=O\left(G^{3}\right),$$ (20)

$$n_{\text{min}}=G+1=O(G).$$ (21)

The group-symmetric model is thus significantly more constrained than the unstructured model, with the number of parameters growing polynomially with *G* instead of exponentially (compare Eq 3). Note how *p*_eff_ < *p* (when *G* ≥ 3), a reduction in the effective number of dimensions that was not apparent from $\Sigma_{G}$ alone.

Nevertheless, even with these symmetry constraints, *n*_min_ still grows with *G*, albeit linearly (Eq 21) instead of exponentially (Eq 3). This means that, for a fixed set of *n* replicates, there will always be a limit to the number of generations that can be analyzed. We need an additional constraint.

#### Sparsity

The additional constraint comes from imposing a sparsity requirement on each irreducible subspace by restricting the Markov order, M, of each time series. This constraint represents a simple case of a decomposable graphical model and results in a Markov-constrained spectral covariance estimate, ${\hat{\Sigma }}_{\Omega }$ and structured covariance estimate ${\hat{\Sigma }}_{G}$. As shown in Section S1.3 in S1 Appendix, the minimum number of replicates required to ensure positive definiteness is now $n_{\text{min}}=M+2$, which, as desired, is independent of *G*. Thus, if M=1 for example, only *n* ≥ 3 pedigrees are required to ensure $\hat{\Sigma }$ is positive definite, regardless of the number of generations analyzed.

#### Missing data

The covariance estimates described above assume complete data. In reality, some measurements are missing, often because data collection is imperfect but also because cells die and have no descendants (although in the datasets analyzed in the paper, cell death is essentially negligible). A simple solution is to apply the Expectation-Maximization (EM) algorithm [73], assuming a multivariate Gaussian to impute the missing data. This is described in Section S1.4 in S1 Appendix.

We remark that, until now, the covariance estimation procedure we have described is distribution-free, providing a non-parametric estimate of second-order variation. It is only to account for missing data that we invoke a distributional assumption. In Section S1.5 in S1 Appendix we show that the maximum likelihood estimate (MLE) for a multivariate Gaussian with the symmetry and Markovian constraints discussed above is in fact the covariance estimate we have already found.

#### Summary of algorithm

Here we summarize the complete algorithm for estimating the mean and covariance of a complete tree, showing how the symmetry and sparsity constraints are incorporated into the EM algorithm to account for missing data:

1. Initialize $\hat{\mu }$ and $\hat{\Sigma }$ with a starting guess.
2. *Expectation step*. Estimate the expected value of the sufficient statistics for each replicate by accounting for missing data using the current estimates for $\hat{\mu }$ and $\hat{\Sigma }$ in Eqs S13, S14. Calculate the resulting (unstructured) sample mean, $\bar{y}$, and covariance, ***S***, using Eq S15.
3. *Maximization step*.
  - Impose the symmetry constraint by pooling over shared elements: ${\bar{y}}_{G}=P\left(\bar{y}\right)$ and $S_{G}=P\left(S\right)$ (Eqs 9 and 10). The estimated mean is then $\hat{\mu }={\bar{y}}_{G}$.
  - Transform to the sample spectral covariance, $S_{\Omega }=T^{†}S_{G}T$.
  - From each unique irreducible block, $S_{\Omega }^{\left(\ell \right)}$, estimate ${\hat{\Sigma }}_{\Omega }^{\left(\ell \right)}$ using S9 and S10, assuming a Markov chain of order M.
  - Construct the spectral covariance estimate ${\hat{\Sigma }}_{\Omega }$ from a direct sum of irreducible blocks ${\hat{\Sigma }}_{\Omega }^{\left(\ell \right)}$ (Eq S11).
  - Inverse transform to the structured covariance estimate, ${\hat{\Sigma }}_{G}=T{\hat{\Sigma }}_{\Omega }T^{†}$. This is the new covariance estimate, $\hat{\Sigma }={\hat{\Sigma }}_{G}$.
4. Return to Step 2 until convergence.

## Results

Our method for estimating $\hat{\Sigma }$ described above can in principle be applied to lineages with any number of generations and needs only a few replicates (pedigrees) to ensure positive definiteness. We now turn to the problem of interpreting $\hat{\Sigma }$ and using it to answer questions about a lineage.

### Lineage variability maps

We can visualize ${\hat{\Sigma }}_{G}$ and ${\hat{\Sigma }}_{\Omega }$ using graphical models to produce different ‘maps’ of the variation in the lineage. We call these lineage variability maps. To depict ${\hat{\Sigma }}_{G}$ we use undirected graphs, since lineal positions within a generation have no ordering. We call the result a lineage correlation map. To depict ${\hat{\Sigma }}_{\Omega }$ we can use directed graphs, since the natural variables in an irreducible subspace are ordered in a sequence. *Thus the spectral transformation enables the undirected graph to be converted into a directed one*. This graph, which we call a dynamic lineage map, compactly represents the dynamics of the bifurcated expression pattern in each subtree.

#### Lineage correlation map

To visualize the network of statistical associations between different lineal positions we use undirected graphs [74, 75] defined either by marginal or by conditional associations. For the network of marginal associations the strength of an edge between a pair of variables is defined by the Pearson correlation coefficient, $\rho_{jj^{\prime }}=\sigma_{jj^{\prime }}/\sqrt{\sigma_{jj}\sigma_{j^{\prime }j^{\prime }}}$ where *σ*_*jj*′_ is an element of $\hat{\Sigma }$. For the network of conditional associations the strength of an edge is determined by the partial correlation $\varrho_{jj^{\prime }|V\backslash \left\{j,j^{\prime }\right\}}=-\kappa_{jj^{\prime }}/\sqrt{\kappa_{jj}\kappa_{j^{\prime }j^{\prime }}}$ where *κ*_*jj*′_ is an element of $\hat{K}$, and *V*∖{*j*, *j*′} refers to the set of variables excluding *j* and *j*′.

**Fig 9:**
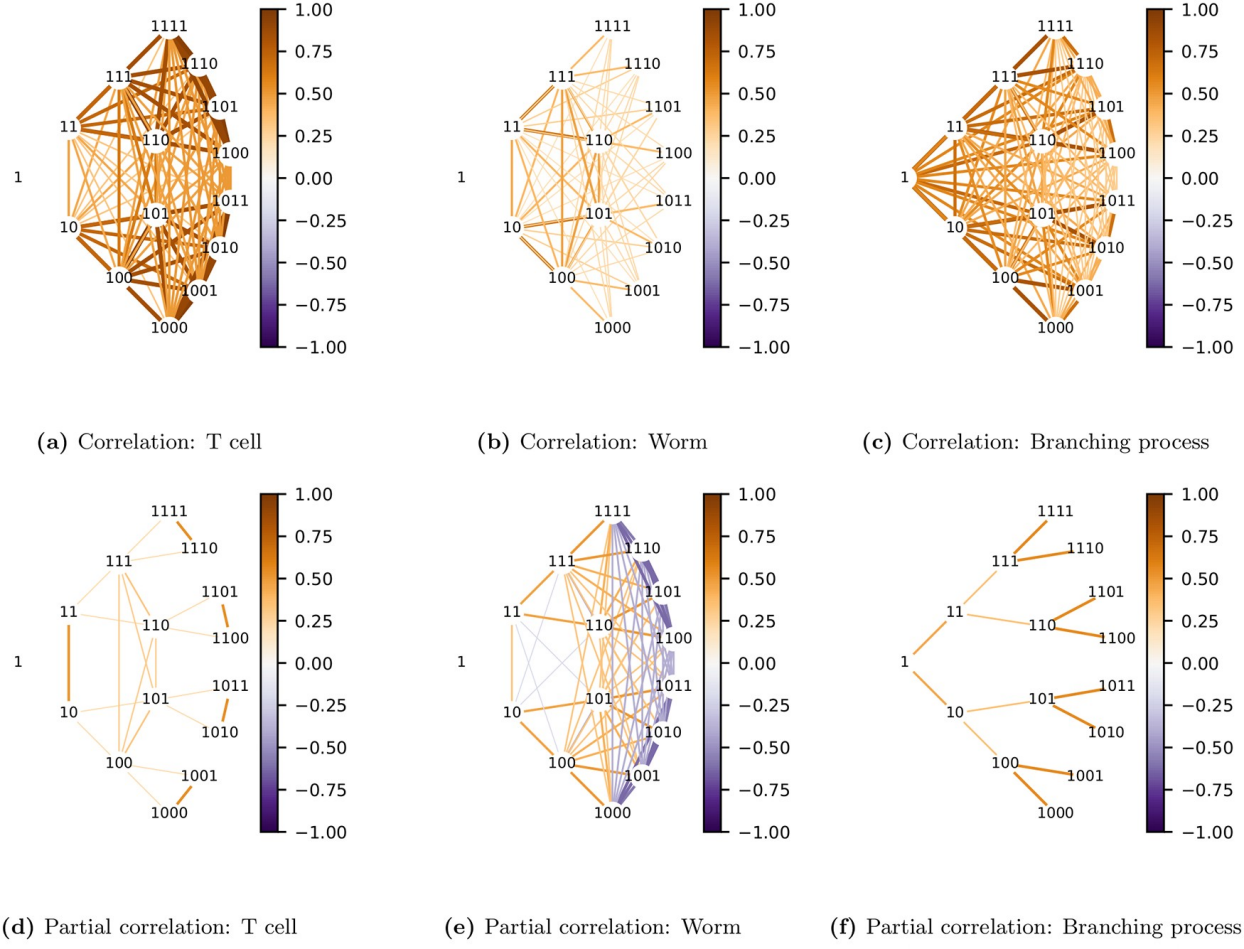
Lineage correlation maps. These are undirected graphs in the original variables (shown as binary numbers). Each generation is arranged in an arc centered on the root node. The color of edges in each graph corresponds to the correlation (top row) or partial correlation (bottom row) between pairs of lineal positions. To avoid clutter, only the first 4 generations are shown. Note how the graph (f) of partial correlations for the simulated branching process, where daughters are conditionally uncorrelated, is a binary tree. This is not the case for the real lineages.

Both types of undirected graphs are shown in Fig 9 for the 3 lineage types. The network of conditional associations identifies direct interactions between variables, conditioned on all other variables, and, as expected, generally provides a sparser representation than does the network of marginal associations.

Note how a binary tree is revealed in the graph of partial correlations for the branching process (Fig 9f). This is expected since our branching process defined daughters to be conditionally uncorrelated. In the network of partial correlations this assumption reveals itself as the lack of an edge between sisters. In contrast, in the partial correlation graphs for T-cell (Fig 9d) and worm (Fig 9e) lineages, sisters are often joined by edges. This arises when the correlation between sisters is greater or less than the squared correlation between mother and daughter, a long-documented observation in cell lineages (see e.g. [28, 29]). This is the simplest demonstration of the fact that phenotypic variation in real lineages cannot be modeled as a branching process.

The graphs in Fig 9 allow us to examine how the network of *phenotypic* associations compares with the network of *lineal* relationships; though the latter is a binary tree, the former may not be. This emphasizes that, although we must assume that phenotypic variation in an unordered tree has the *symmetry* of a binary tree, we do not assume it has the *sparsity* of a binary tree.

A problem with representing each lineal position as a node is that the graph appears cluttered since there are many edges and nodes with similar strengths. This problem gets exponentially worse with increasing generations. Such redundancies disappear when examining the tree over its natural variables.

#### Dynamic lineage map

The spectral transformation dramatically simplifies the lineage variability map, decomposing the (potentially) complete graph over all lineal positions shown in the previous section into a set of *G* independent graphs, each of which is a time series. Since the natural variables in an irreducible subspace have a clear ordering (by generation), an irreducible block can be represented by a directed graph [76–78], with each of its variables conditioned on the past only (rather than on both the past and the future).

To construct such a directed graph, consider the vector of natural variables, ***z***_*ℓ*_, belonging to each irreducible subspace, *ℓ*. The irreducible block is then given by

$$\begin{array}{c} \Sigma_{\Omega }^{\left(\ell \right)}=E\left(z_{\ell }z_{\ell }^{\prime }\right). \end{array}$$ (22)

Since the variables in ***z***_*ℓ*_ comprise a time series, they can be modeled using a linear structural equation:

$$\begin{array}{cc} z_{\ell j} & =\varepsilon_{\ell j}+\sum_{j^{\prime }=\ell +1}^{j-1}\beta_{\ell jj^{\prime }}z_{\ell j^{\prime }},\;\text{for}\;\ell <j\leq G, \end{array}$$ (23)

where, for a given irreducible subspace *ℓ*, *z*_*ℓj*_ is the natural variable for generation *j*, *β*_*ℓjj*′_ is the regression coefficient of generation *j* on *j*′, and *ε*_*ℓj*_ is an independent random variable describing variation originating at generation *j* that has a mean of zero and expected variance $E(\varepsilon_{\ell j}^{2})$. To determine these model parameters, we start by rewriting Eq 23 in terms of a lower-triangular matrix ***B***_*ℓ*_ = (*b*_*ℓjj*′_):

$$B_{\ell }z_{\ell }=\varepsilon_{\ell },$$ (24)

$$\begin{array}{cc} b_{\ell jj^{\prime }} & =\{\begin{array}{cc} 1, & \text{if}\;j=j^{\prime }\\ 0, & \text{if}\;j-j^{\prime }<0\;\text{or}\;j-j^{\prime }>M\\ -\beta_{\ell jj^{\prime }}, & \text{otherwise}. \end{array} \end{array}$$ (25)

Now, the modified Cholesky decomposition of $\Sigma_{\Omega }^{\left(\ell \right)}$ is

$$\begin{array}{c} \Sigma_{\Omega }^{\left(\ell \right)}=L_{\ell }\Phi_{\ell }L_{\ell }^{\prime }, \end{array}$$ (26)

where **Φ**_*ℓ*_ is diagonal and ***L***_*ℓ*_ is lower triangular. Combining this with Eq 22 and then Eq 24, we see that

$$L_{\ell }\Phi_{\ell }L_{\ell }^{\prime }=E(z_{\ell }z_{\ell }^{\prime }),$$ (27)

$$=B_{\ell }^{-1}E\left(\varepsilon_{\ell }\varepsilon_{\ell }^{\prime }\right){\left(B_{\ell }^{-1}\right)}^{\prime }.$$ (28)

Thus the parameters in the structural equation model are found directly from the modified Cholesky decomposition:

$$\begin{array}{cc} B_{\ell } & =L_{\ell }^{-1},\;E\left(\varepsilon_{\ell }\varepsilon_{\ell }^{\prime }\right)=\Phi_{\ell }. \end{array}$$ (29)

The directed graph can then be defined with edge weights given by *β*_*ℓjj*′_ and node sizes given by $E(\varepsilon_{\ell j}^{2})$. The edge weights represent transmission of variation while the node sizes represent innovations. If |*β*_*jj*′_|<1 then transmission is regressive, with descendants gradually losing memory of previous generations. However, if |*β*_*jj*′_|>1 then variation from source (*ℓ*) observed at generation *j*′ is *amplified* during transmission to generation *j*. Thus large variation can either arise directly from a large innovation or it can be the result of strong amplification of small variation (or both).

**Fig 10:**
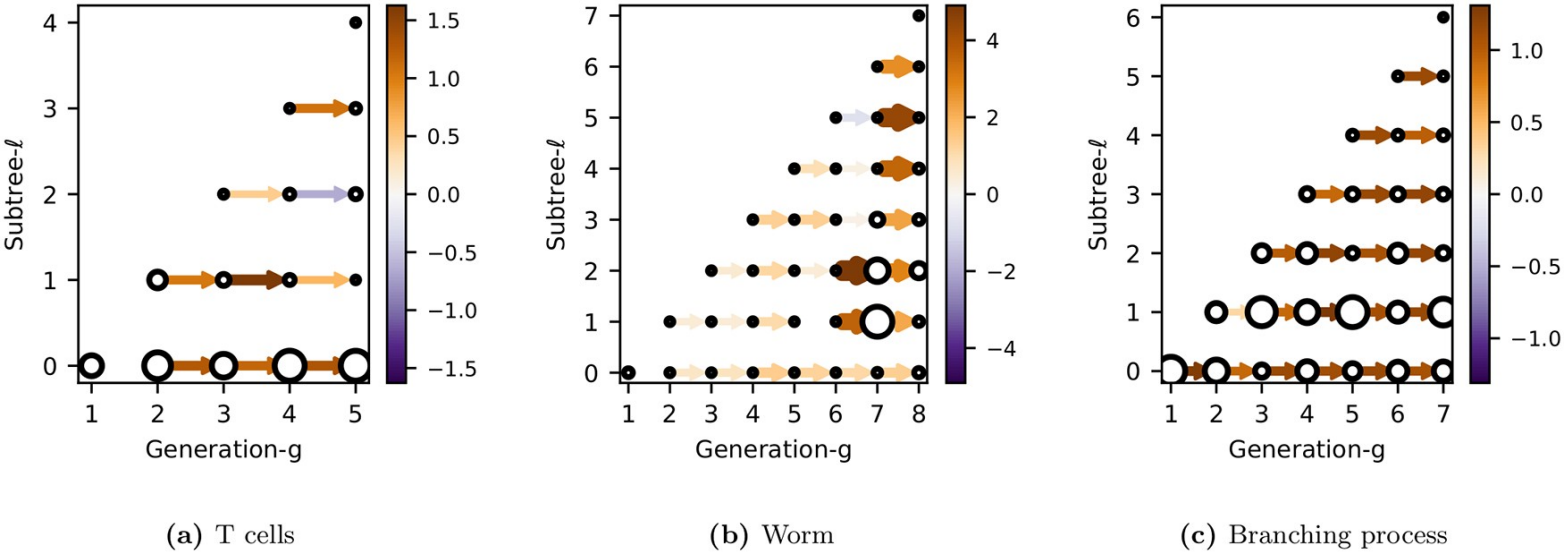
Dynamic lineage maps. These directed graphs in the natural variables show the dynamics of the bifurcated expression pattern in each subtree *ℓ*. The color (and thickness) of an edge between node *j* and *j*′ corresponds to the transmission strength, *β*_*ℓjj*′_. The size of the node corresponds to the innovation strength, $E(\varepsilon_{\ell j}^{2})$.

These directed graphs compactly summarize the dynamics of phenotypic variation along each subtree. Examples for the 3 lineages types are shown in Fig 10. Each connected component, given by a row, represents how the bifurcated expression pattern associated with a subtree *ℓ* propagates down successive generations.

As expected, the worm graph has the most distinct structure (Fig 10b). Transmission and innovation is small for the first few generations of each subtree, before “turning on” after generation 6. This means that the bifurcated expression of a subtree is silent for many generations before appearing simultaneously in multiple descendants at a later generation. Note how transmission and innovation for *ℓ* = 0 are weak, indicating little inter-pedigree variation, as expected for a totipotent cell. Strong transmission is observed in particular subtrees at certain generations. For example, transmission is highest for *ℓ* = 2 between generations 6 and 7, and for *ℓ* = 5 between generations 7 and 8. We will discuss these features later when we assess the fate restriction associated with each subtree.

Although, for the worm, these characteristics could have been inferred just by visualizing the pedigrees directly, the point is that we now have a statistical method to extract such features when the lineage is variable. For example, the primary feature of the graph for T cells, which was not obvious from just looking at the lineages, is that subtree *ℓ* = 0 has the largest innovations and consistently strong transmission between generations (the exception is from generation 1, whose phenotype is not transmitted). This indicates that much of the variation is between pedigrees, rather than within the pedigree as it was for the worm. We will describe this in more detail in the next section.

Finally, we note that the graph for the branching process is essentially featureless across all generations and in all subtrees, as would be expected for a stationary process.

### Fate profiles

Lineage variability maps describe the pattern of phenotypic associations throughout the lineage. However, as with lineage maps, our interest is often in using them to infer where fate is specified. In the introduction, we described how this involves identifying the most recent common ancestor of cells with shared fate. For a clonal pattern, where a cell fate is exclusive to a single subtree, we would infer that fate was specified at (or near) a single lineal position—the root of that subtree. For a non-clonal pattern, where cell fate is expressed in multiple subtrees, we would infer that fate was specified at multiple lineal positions. In *C. elegans* these inferences can be made visually [5]. Here we show how, by knowing the lineage variability map **Σ**, we can make these inferences statistically, overcoming the problem of how to identify subtrees with shared phenotypes when lineages are variable.

Before we begin, we must define what we mean by cell fate. In this study we define cell fate to be the measured phenotype of a cell at the latest generation studied, *G*. This practical definition allows us to analyze cell fate whether or not the phenotype in the last generation is actually a terminal fate. Also, by defining cell fate as the phenotype itself rather than as a cell type (determined by that phenotype), we can use the phenotypic measurements as is, without having to cluster or threshold them. Such discretization procedures can be difficult to define when phenotypes exist on a continuum of differentiation, as is often the case [79].

Having defined fate, we turn now to explaining its variability in terms of aspects of the lineage. We first partition the variability among the subtrees, or sources of variation. This quantifies how much of a cell’s fate is restricted by, or specified by, each subtree. We then examine the correlation of a cell’s fate with the phenotypes of its ancestors. This identifies the earlier generations over which a phenotypic fate has been stably expressed. Together these two measures, of fate restriction and fate expression, make up what we call fate profiles.

#### Fate restriction by subtree

To determine how much cell fate is restricted by (i.e. specified by) each subtree, we partition the fate variability among the different sources of variation, each of which is located at the root of a *bifurcated* subtree. This is just the traditional problem of variance components analysis in nested groups (see Fig 5b). Since we have already calculated the spectral covariance matrix, we need only locate the appropriate components of variance along its diagonal (see Eq 18).

Consider the variance of a cell in generation *G*, given by *σ*_*GGG*_ (see Eq 5). This can be written as the the sum of independent contributions from each source (*ℓ*, *τ*). These are known as the (normalized) components of variance in a classical ANOVA [71]. A convenient way to show this decomposition in our framework is to perform the inverse spectral transform of **Σ**_Ω_ (for an example, see Section S1.2.9 in S1 Appendix). The result is

$$\begin{array}{cc} \sigma_{GGG} & =\frac{1}{N_{\text{src}}}\sum_{\ell =0}^{G-1}\sum_{\tau =0}^{d_{\ell }-1}\xi_{GG}^{\left(\ell \right)},\;\;d_{\ell }=\{\begin{array}{cc} 1, & \text{if}\;\ell =0,\\ 2^{\ell -1}, & \text{if}\;\ell \geq 1, \end{array} \end{array}$$ (30)

$$\begin{array}{c} =\frac{1}{N_{\text{src}}}\sum_{\ell =0}^{G-1}\xi_{GG}^{\left(\ell \right)}d_{\ell }, \end{array}$$ (31)

where *d*_*ℓ*_ is the number of transverse sources of variation at a given *ℓ*, and $N_{\text{src}}=\sum_{\ell =0}^{G-1}d_{\ell }=2^{G}$ is the total number of sources of variation in a *G*-generation tree. The component of variance corresponding to source *ℓ* is thus given by $\xi_{GG}^{\left(\ell \right)}d_{\ell }/N_{\text{src}}$ where $\xi_{GG}^{\left(\ell \right)}$ is found along the diagonal of **Σ**_Ω_.

The resulting proportion of variance attributable to the *ℓ*-th source for a cell in generation *G* is given by

$$\begin{array}{c} \eta^{2}\left(\ell |G\right)=\frac{\xi_{GG}^{\left(\ell \right)}d_{\ell }}{\sum_{\ell^{\prime }=0}^{G-1}\xi_{GG}^{\left(\ell^{\prime }\right)}d_{\ell^{\prime }}},\;0\leq \ell <G. \end{array}$$ (32)

This measures the relative importance of each source of variation *ℓ* in explaining cell fate. Equivalently, it measures how much cell fate is restricted by subtree *ℓ*.

It will also be useful to calculate the cumulative proportion of total variance attributable to subtrees from 0 to *ℓ*, inclusive,

$$\begin{array}{c} \eta_{\text{cml}}^{2}\left(\ell |G\right)=\frac{\sum_{\ell^{\prime }=0}^{\ell }\xi_{GG}^{\left(\ell^{\prime }\right)}d_{\ell^{\prime }}}{\sum_{\ell^{\prime }=0}^{G-1}\xi_{GG}^{\left(\ell^{\prime }\right)}d_{\ell^{\prime }}},\;0\leq \ell <G. \end{array}$$ (33)

This gives a running total of the cell fate restricted by each successive subtree, starting at *ℓ* = 0 and is related to the intraclass correlation.

An obvious question is whether *η*^2^(*ℓ*|*G*) would differ if we had simply performed a variance components analysis on the single generation *G*, ignoring measurements in the other generations. With complete data, our method would give the identical result to a variance components calculation: using a decomposable model for a Markov chain ensures that estimates of diagonal elements in **Σ**_Ω_ (the components of variance) are given by the corresponding diagonal elements in ***S***_Ω_. If there were incomplete data however, data from other generations would help to estimate the missing data in generation *G*, improving the estimate of *η*^2^(*ℓ*|*G*).

#### Fate expression by generation

Having determined how much fate is restricted by each subtree, we now determine how much cell fate is expressed in each earlier generation. We do this by correlating the phenotype of a cell in generation *G* with those of its direct ancestors. The degree to which earlier generations are correlated with the last is a measure of when fate becomes expressed.

This definition of fate expression emphasizes the stability, or persistence, of a phenotypic fate rather than the absolute value of a phenotypic measurement. We have chosen this definition since our analysis should be general enough to work on data with substantial variability, where it may be difficult to define a cell fate in terms of some threshold level of expression.

Given a lineal position in generation *G* and its direct ancestor in generation *g*, the proportion of explained variance is just the squared correlation coefficient, or coefficient of determination,

$$\begin{array}{c} R^{2}\left(g|G\right)=\frac{\sigma_{gG}^{2}}{\sigma_{gg}\sigma_{GG}}=\rho_{gG}^{2},\;1\leq g<G. \end{array}$$ (34)

In the subscripts we have simplified the 3-index notation from Eq 5 by ignoring the third index. This does not cause confusion since in this context we are only concerned with direct ancestors.

Generalizing to prediction using multiple generations of direct ancestors up to and including that in generation *g* gives

$$\begin{array}{c} R_{\text{cml}}^{2}\left(g|G\right)=\frac{\Sigma_{Gg}\Sigma_{gg}^{-1}\Sigma_{gG}}{\sigma_{GG}} \end{array}$$ (35)

where ***g*** represents a vector of direct ancestors of the cell in generation *G* that are from generations 1 to *g* inclusive. Note that Eq 35 accounts for possible dependencies in the variation between ancestors. Unlike for the case of components of variance, contributions from different ancestral generations are not (in general) orthogonal.

#### Comparing fate restriction and fate expression

**Fig 11:**
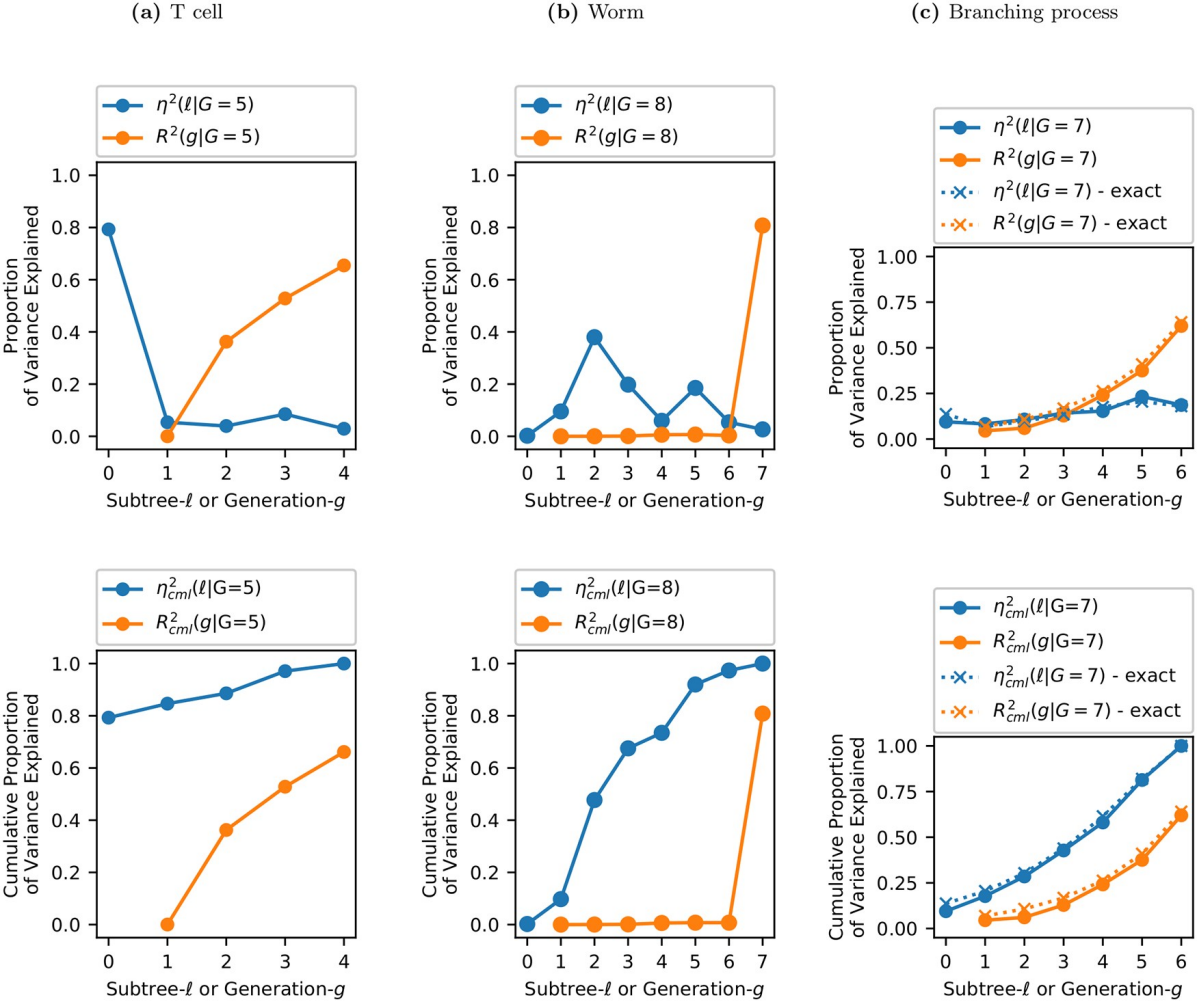
Fate profiles for different lineages. Explained variance (top row) and the cumulative explained variance (bottom row). *η*^2^(*ℓ*|*G*) (blue) measures how much the fate of a cell at generation *G* is restricted by each subtree *ℓ*. *R*^2^(*g*|*G*) (orange) measures how much a generation-*G* cell’s phenotype is correlated with its direct ancestor in generation *g*. Note that because the Markov process is assumed to be first order (see Section ‘Sparsity.’), $R^{2}=R_{\text{cml}}^{2}$. For the case of the simulated branching process the exact result is also shown. This illustrates the accuracy of the inference procedure.

Our measures of fate restriction and fate expression are complementary ways of explaining the variation of cell fate: *η*^2^(*ℓ*|*G*) explains fate in terms of shared ancestry (subtrees) while *R*^2^(*g*|*G*) explains fate in terms of ancestral phenotypes. We call these fate profiles. Both are plotted in Fig 11, with the top row giving the explained variance and the bottom row giving the cumulative explained variance.

*η*^2^(*ℓ*|*G*) (blue line, top row) shows how much variation in *G* is restricted by each of the subtrees *ℓ*. For T cells, *ℓ* = 0 is by far the most important “subtree” for explaining fate (at *G* = 5). This is consistent with a cell that has limited potency, where the choice of founder cell severely restricts the range of fates available. In this case, any founder cell has already had 80% of its cell fate restricted. For the worm, cell fate is restricted by all subtrees *except* *ℓ* = 0. Each zygote thus has 100% of its cell fate potential. This is consistent with the behavior for a totipotent cell. All subsequent subtrees contribute to cell fate, with *ℓ* = 2, 3, 5 being particularly important. This spread of fate specification over different subtrees might have been expected given the non-clonal expression pattern of *PHA-4*. While a clonal pattern is projected onto a single subtree, non-clonal patterns are projected onto multiple subtrees. For the branching process, contributions from all subtrees are comparable, as expected. Each subtree is, roughly speaking, equally important.

*R*^2^(*g*|*G*) (orange line, top row) gives the correlation of a cell in generation *G* with its direct ancestor in generation *g*. For T cells, *R*^2^(*g*|*G*)≃0 for *g* = 1 indicating that, even though most cell fate (at least at *G* = 5) is set by the choice of founder cell, the founder does not actually resemble its descendants. For the worm, *R*^2^(*g*|*G*)≃0 for 1 ≤ *g* ≤ 6. Thus none of the complicated structure in *η*^2^ for 0 ≤ *ℓ* ≤ 6 is reflected in *R*^2^.

This difference between fate restriction and fate expression is highlighted by the cumulative explained variance shown in the bottom row of Fig 11. For the worm, $\eta_{\text{cml}}^{2}$ increases with each subtree (for *ℓ* > 0) while $R_{\text{cml}}^{2}\left(g|G\right)$ remains zero until *g* = 7. For the T cell, $\eta_{\text{cml}}^{2}$ starts high at *ℓ* = 0, while $R_{\text{cml}}^{2}\left(g|G\right)$ starts at zero and increases slowly with each generation. Contrast this with the branching process where $\eta_{\text{cml}}^{2}$ and $R_{\text{cml}}^{2}$ both start near zero and increase steadily in a similar fashion. Clearly a T cell lineage cannot be modeled as a branching process.

In the worm lineage, such *fate restriction before fate expression* captures what is perhaps obvious from the lineage map. Looking at Fig 1b we see how *PHA-4* expression is negligible until generation 7 whereupon it appears simultaneously across multiple subtrees. This implies that cells across those subtrees coordinated their fates before expressing them. Thus, for the worm, the fate profile merely restates, albeit in a quantitative way, what can be visualized in a single (invariant) pedigree. However, the advantage of the fate profile is that it can be applied to variable lineages, when simple visualization fails.

## Discussion

The lineage map, which has been instrumental in the discovery of fate specification mechanisms in simple organisms, was born from the study of invariant lineages and is not a particularly useful concept for understanding the more ubiquitous case of variable lineages. To address this limitation, we have introduced lineage variability maps, which provide a way to describe lineages at the population level. Whereas the lineage map is a description of the fixed pattern of phenotypes across a pedigree, the lineage variability map describes the pattern of phenotypic associations across a pedigree. This map of phenotypic associations, **Σ**, provides quantitative answers to essential scientific questions such as those about cell potency, fate restriction, and the sources of variation in a lineage.

We have constructed lineage variability maps from a sample of highly-variable pedigrees from CD8^+^ T-lymphocytes up to five generations. These show that most of the variation in cell fate, defined here to be average cell size at generation 5, is explained by the choice of naive cell. Yet, despite the pivotal role played by this founder in restricting cell fate, its phenotype is not predictive of fate: though a naive cell may specify that its descendants be large, it may not be large itself (at least on average).

Although we expect to apply our technique primarily to variable pedigrees which are difficult to interpret by visualization alone, we can also apply it to invariant lineages to check our results. In fact, by constructing lineage variability maps from sample wild-type pedigrees from *C. elegans* marked for pharyngeal expression, we successfully recovered essential information in the known lineage map, identifying global features such as the small degree of inter-pedigree variation characteristic of a totipotent zygote, and the several-generation delay between fate specification and expression.

Yet our lineage variability maps capture important finer detail as well. Consider the peak in fate restriction at *ℓ* = 2 observed in Fig 11b. This arises from the strong bifurcation of fate traced back to the division of both P1 and of AB, progenitors located at *ℓ* = 2 (see Section S1.6 in S1 Appendix for the labeling of lineal positions in the worm). That only a single daughter from P1 and from AB exhibit pharyngeal fate results in the spike in fate restriction that we observe. Interestingly, this phenomenon, of pharyngeal fate ensuing from two cousins at generation 3 (ABa and EMS) but not from their sisters, is a phenomenon that has been investigated in detail [80]. Such work laid the foundation for further studies leading to the understanding of the molecular and cellular mechanisms for specification of pharyngeal tissue [81]. This demonstrates how, even though we may be ignorant of the ordering of the lineage, we can still detect a fate bifurcation event of biological relevance that had previously required knowledge of this ordering. In other words, although we must assume lineage relationships are symmetric, this does not prevent us from detecting the effects of asymmetric lineage patterns from the ‘boost’ they give to the variance in particular subtrees.

Recent technological innovations have introduced a variety of methods for recording lineage data, involving both advanced imaging [19, 46–48] and genetic barcoding [20, 49, 51–57] techniques. With the statistical lineage mapping and fate profiling methods described in this manuscript, it should be possible to quantify fundamental properties of these lineages, such as the potency of progenitors, whether heterogeneity is clonal, and at what depth such heterogeneity appears. Just as the visual identification of fate bifurcations in the worm lineage map enabled the location of fate specification events to be discovered, the capacity to perform systematic screens to rapidly identify the important stages of fate restriction should contribute to a deeper understanding of the mechanisms of fate specification in more complex, more variable systems.

## Supporting information

S1 Appendix Supplemental derivations and nomenclature. (PDF)

S1 Data Lineage data. (XLSX)
