# Supplementary material for: Maps of variability in cell lineage trees
Source: PLoS Comput Biol. 2019 Feb 12;15(2):e1006745. doi: 10.1371/journal.pcbi.1006745 (PMC6388934; doi:10.1371/journal.pcbi.1006745)
Supplement: S1 Appendix — (PDF) [file pcbi.1006745.s001.pdf]

S1 Appendix

Supplemental derivations and nomenclature.

S1.1 Group symmetry and matrix decomposition

Here we summarize a fundamental result of group representation theory: how the vector space for a matrix that is invariant under a symmetry group can be decomposed into subspaces that do not interact with each other. We follow the procedure described in [1, 2] for the general case. In the next section we apply it to a binary tree.

Let $\vartheta : s \to \boldsymbol{D}_s \ \forall s \in \mathcal{G}$ be the representation of the group $\mathcal{G}$ on $\mathbb{R}^p$. From Maschke's theorem, and by induction, a linear representation ϑ of a finite group is a direct sum of irreducible representations. Accordingly, $\vartheta = \bigoplus_{\omega=1}^{\mathcal{I}} m_\omega \vartheta^{(\omega)}$ where each inequivalent irreducible representation $\vartheta^{(\omega)}$ has a multiplicity m_ω and a dimensionality d_ω such that $p = \sum_{\omega=1}^{\mathcal{I}} m_\omega d_\omega$. This ensures that any matrix $\boldsymbol{M} \in \mathcal{W}_{\mathcal{G}}$, where $\mathcal{W}_{\mathcal{G}}$ is the set of matrices invariant under $\mathcal{G}$, can be decomposed as

$$\boldsymbol{T}^\dagger \boldsymbol{M} \boldsymbol{T} = \begin{pmatrix} \mathcal{C}^{(1)} & & 0 \\ & \ddots & \\ 0 & & \mathcal{C}^{(\mathcal{I})} \end{pmatrix}, \quad \boldsymbol{M} \in \mathcal{W}_{\mathcal{G}}, \tag{S1}$$

where each $\mathcal{C}^{(\omega)} \in \mathbb{R}^{m_\omega d_\omega \times m_\omega d_\omega}$ is associated with an isotypic subspace. Here $\boldsymbol{T}$ is a unitary change-of-basis matrix that transforms $\boldsymbol{M}$ to a symmetry-adapted basis where its block diagonal form is revealed. $\boldsymbol{T}$ is defined by the symmetry group of $\boldsymbol{M}$.

Furthermore, Schur's lemma states that, since $\mathcal{C}^{(\omega)}$ and ϑ_ω commute, the isotypic subspaces themselves decompose and can be written as a direct sum of repeated subblocks,

$$\mathcal{C}^{(\omega)} = \begin{pmatrix} M_\Omega^{(\omega)} & & 0 \\ & \ddots & \\ 0 & & M_\Omega^{(\omega)} \end{pmatrix} \tag{S2}$$

where there are d_ω repeated subblocks of $M_\Omega^{(\omega)} \in \mathbb{R}^{m_\omega \times m_\omega}$. Note that this requires that $\boldsymbol{T}$ be specified with the appropriate arrangement of symmetry-adapted basis vectors in each isotypic subspace (see p.40-43 [1]).

The fundamental decomposition of $\boldsymbol{M} \in \mathcal{W}_{\mathcal{G}}$ is thus

$$\begin{aligned} \boldsymbol{T}^\dagger \boldsymbol{M} \boldsymbol{T} &= \bigoplus_{\omega=1}^{\mathcal{I}} \left[\bigoplus_{\nu=1}^{d_\omega} M_\Omega^{(\omega)} \right], \\ &= \bigoplus_{\omega=1}^{\mathcal{I}} \left[I_{d_\omega} \otimes M_\Omega^{(\omega)} \right], \quad M_\Omega^{(\omega)} \in \mathbb{R}^{m_\omega \times m_\omega}, \end{aligned} \tag{S3}$$

where ν indexes the repeated subblocks $M_\Omega^{(\omega)}$ and I_{d_ω} is the identity matrix of dimension d_ω. This expression highlights how the $M_\Omega^{(\omega)}$ are orthogonal building blocks of $\boldsymbol{M}$. We refer to each $M_\Omega^{(\omega)}$ as an irreducible block. Each irreducible block describes the interactions between m_ω natural variables which span what we call an irreducible subspace. Irreducible blocks are thus the main objects of inference and, as we will see for the case of a binary tree, are each associated with a source of variation.

Eq S3 states that a p-dimensional space decomposes into $\mathcal{I}$ *unique* irreducible subspaces (each repeated d_ω times) each with m_ω dimensions. This reduces the number

of pairwise associations in the model since Eq S3 only permits associations between variables *within* an irreducible subspace. It also reduces the total number of unique variables since only one of the d_ω identical copies of each irreducible subspace needs to be considered. Note that when $d_\omega > 1$, which can occur only for a non-commutative group, Schur's lemma states that degenerate eigenvalues are present. Degeneracy reduces the number of variables, and thus the dimensionality, of the problem. For the binary tree degeneracy occurs when the number of generations is 3 or higher.

S1.2 Group representation for a complete tree

The finite group symmetry of a matrix refers to the set of permutations of variables that keep the matrix invariant. As described in the previous section, this invariance property defines a set of orthogonal subspaces of the original vector space. Formal derivation of the orthogonal subspaces for a particular group symmetry representation follows a standard procedure [1]. Since this does not appear to have been done for a representation on a complete tree we give a detailed derivation here, for binary trees with 2 and 3 generations. Generalization of the key results to higher generations then becomes apparent.

S1.2.1 Symmetry groups

The abstract symmetry group of the tree is set by the largest generation being studied. In general the group involves recursions of wreath products.

Generations 1,2: With two generations the symmetry group is just the $\mathcal{S}_2$ group, the cyclic group containing 2 elements:

$$\mathcal{G}_2 \sim \mathcal{S}_2. \tag{S4}$$

Generations 1,2,3: The symmetry group of a binary tree containing the first 3 generations is given by

$$\begin{aligned}\mathcal{G}_3 &\sim (\mathcal{S}_2 \times \mathcal{S}_2) \rtimes \mathcal{S}_2\\ &\sim \mathcal{S}_2 \wr \mathcal{S}_2\\ &\sim \mathcal{D}_4\end{aligned}$$

where $\times$ is the direct product, $\rtimes$ is the semi-direct product and $\wr$ is the wreath product. This group has order 8 and is isomorphic to the dihedral group $\mathcal{D}_4$, the symmetry group for a square.

S1.2.2 Permutation representation

The permutation matrix representations, $\boldsymbol{D}(s)$, that leave the covariance matrix invariant can be found manually. Here they are shown divided up into equivalence classes. $\chi[\boldsymbol{D}(s)]$ is the character of each class. Note that dots are used to represent 0 when the matrix element corresponds to a pair of cells from two different generations (which we assume can not be permuted since variation is not stationary between generations).

Generations 1,2:

Class I: $s = \{e\}$, $\chi[\boldsymbol{D}(s)] = 3$:

$$\boldsymbol{D}(e) = \begin{pmatrix} 1 & \cdot & \cdot \\ \cdot & 1 & 0 \\ \cdot & 0 & 1 \end{pmatrix},$$

Class II: $s = \{c\}$, $\chi[\boldsymbol{D}(s)] = 1$:

$$\boldsymbol{D}(c) = \begin{pmatrix} 1 & \cdot & \cdot \\ \cdot & 0 & 1 \\ \cdot & 1 & 0 \end{pmatrix}.$$

Generations 1,2,3: The 8 group elements, s, of $\mathcal{G}_3$ are each represented by a 7-dimensional permutation matrix, $D(s)$, corresponding to the 7 positions in a binary tree with 3 generations. These are listed below, grouped into the 5 equivalence classes each with a given character χ:

Class I: $s = \{e\}$; $\chi[\boldsymbol{D}(s)] = 7$:

$$\boldsymbol{D}(e) = \begin{pmatrix} 1 & \cdot & \cdot & \cdot & \cdot & \cdot & \cdot \\ \cdot & 1 & 0 & \cdot & \cdot & \cdot & \cdot \\ \cdot & 0 & 1 & \cdot & \cdot & \cdot & \cdot \\ \cdot & \cdot & \cdot & 1 & 0 & 0 & 0 \\ \cdot & \cdot & \cdot & 0 & 1 & 0 & 0 \\ \cdot & \cdot & \cdot & 0 & 0 & 1 & 0 \\ \cdot & \cdot & \cdot & 0 & 0 & 0 & 1 \end{pmatrix},$$

Class II: $s = \{c^2\}$, $\chi[\boldsymbol{D}(s)] = 3$:

$$\boldsymbol{D}(c^2) = \begin{pmatrix} 1 & \cdot & \cdot & \cdot & \cdot & \cdot & \cdot \\ \cdot & 1 & 0 & \cdot & \cdot & \cdot & \cdot \\ \cdot & 0 & 1 & \cdot & \cdot & \cdot & \cdot \\ \cdot & \cdot & \cdot & 0 & 1 & 0 & 0 \\ \cdot & \cdot & \cdot & 1 & 0 & 0 & 0 \\ \cdot & \cdot & \cdot & 0 & 0 & 0 & 1 \\ \cdot & \cdot & \cdot & 0 & 0 & 1 & 0 \end{pmatrix},$$

Class III: $s = \{c, c^{-1}\}$, $\chi[\boldsymbol{D}(s)] = 1$:

$$\boldsymbol{D}(c) = \begin{pmatrix} 1 & \cdot & \cdot & \cdot & \cdot & \cdot & \cdot \\ \cdot & 0 & 1 & \cdot & \cdot & \cdot & \cdot \\ \cdot & 1 & 0 & \cdot & \cdot & \cdot & \cdot \\ \cdot & \cdot & \cdot & 0 & 0 & 1 & 0 \\ \cdot & \cdot & \cdot & 0 & 0 & 0 & 1 \\ \cdot & \cdot & \cdot & 0 & 1 & 0 & 0 \\ \cdot & \cdot & \cdot & 1 & 0 & 0 & 0 \end{pmatrix},$$

$$\boldsymbol{D}(c^{-1}) = \begin{pmatrix} 1 & \cdot & \cdot & \cdot & \cdot & \cdot & \cdot \\ \cdot & 0 & 1 & \cdot & \cdot & \cdot & \cdot \\ \cdot & 1 & 0 & \cdot & \cdot & \cdot & \cdot \\ \cdot & \cdot & \cdot & 0 & 0 & 0 & 1 \\ \cdot & \cdot & \cdot & 0 & 0 & 1 & 0 \\ \cdot & \cdot & \cdot & 1 & 0 & 0 & 0 \\ \cdot & \cdot & \cdot & 0 & 1 & 0 & 0 \end{pmatrix},$$

Class IV: $s = \{r, c^2 r\}$; $\chi[\boldsymbol{D}(s)] = 5$:

$$\boldsymbol{D}(r) = \begin{pmatrix} 1 & \cdot & \cdot & \cdot & \cdot & \cdot & \cdot \\ \cdot & 1 & 0 & \cdot & \cdot & \cdot & \cdot \\ \cdot & 0 & 1 & \cdot & \cdot & \cdot & \cdot \\ \cdot & \cdot & \cdot & 0 & 1 & 0 & 0 \\ \cdot & \cdot & \cdot & 1 & 0 & 0 & 0 \\ \cdot & \cdot & \cdot & 0 & 0 & 1 & 0 \\ \cdot & \cdot & \cdot & 0 & 0 & 0 & 1 \end{pmatrix},$$

$$\boldsymbol{D}(c^2 r) = \begin{pmatrix} 1 & \cdot & \cdot & \cdot & \cdot & \cdot & \cdot \\ \cdot & 1 & 0 & \cdot & \cdot & \cdot & \cdot \\ \cdot & 0 & 1 & \cdot & \cdot & \cdot & \cdot \\ \cdot & \cdot & \cdot & 1 & 0 & 0 & 0 \\ \cdot & \cdot & \cdot & 0 & 1 & 0 & 0 \\ \cdot & \cdot & \cdot & 0 & 0 & 0 & 1 \\ \cdot & \cdot & \cdot & 0 & 0 & 1 & 0 \end{pmatrix},$$

Class V: $s = \{cr, c^3 r\}$, $\chi[\boldsymbol{D}(s)] = 1$:

$$\boldsymbol{D}(cr) = \begin{pmatrix} 1 & \cdot & \cdot & \cdot & \cdot & \cdot & \cdot \\ \cdot & 0 & 1 & \cdot & \cdot & \cdot & \cdot \\ \cdot & 1 & 0 & \cdot & \cdot & \cdot & \cdot \\ \cdot & \cdot & \cdot & 0 & 0 & 1 & 0 \\ \cdot & \cdot & \cdot & 0 & 0 & 0 & 1 \\ \cdot & \cdot & \cdot & 1 & 0 & 0 & 0 \\ \cdot & \cdot & \cdot & 0 & 1 & 0 & 0 \end{pmatrix},$$

$$\boldsymbol{D}(c^3 r) = \begin{pmatrix} 1 & \cdot & \cdot & \cdot & \cdot & \cdot & \cdot \\ \cdot & 0 & 1 & \cdot & \cdot & \cdot & \cdot \\ \cdot & 1 & 0 & \cdot & \cdot & \cdot & \cdot \\ \cdot & \cdot & \cdot & 0 & 0 & 0 & 1 \\ \cdot & \cdot & \cdot & 0 & 0 & 1 & 0 \\ \cdot & \cdot & \cdot & 0 & 1 & 0 & 0 \\ \cdot & \cdot & \cdot & 1 & 0 & 0 & 0 \end{pmatrix}.$$

S1.2.3 Irreducible representations and character tables

Since $\mathcal{G}_2$ and $\mathcal{G}_3$ are isomorphic to well-known symmetry groups, their irreducible representations can be identified immediately.

Generations 1,2: Since $\mathcal{G}_2 \sim \mathcal{S}_2$, the irreducible representations of $\mathcal{G}_2$ are simply those for $\mathcal{S}_2$. The canonical decomposition of the representation space for a tree can be found by first examining the character table for $\mathcal{G}_2$ (Table A). Here $\Gamma_2^{(\omega)}$ are the irreducible representations of $\mathcal{G}_2$ and ϑ_2 is the completely reducible representation for the 2-generation tree given in the previous section.

	$\{e\}$	$\{c\}$
$\Gamma_2^{(1)}$	1	1
$\Gamma_2^{(2)}$	1	-1
ϑ_2	3	1

Table A. Character table for $\mathcal{G}_2$.

Generations 1,2,3: Since $\mathcal{G}_3 \sim \mathcal{D}_4$ the irreducible representations of $\mathcal{G}_3$ are just those for the symmetry group of the square. The character table is given in Table B where

the $\Gamma_3^{(\omega)}$ are the irreducible representations of $\mathcal{G}_3$ and ϑ_3 is the completely reducible representation for the tree given in Section "Permutation representation".

	$\{e\}$	$\{c^2\}$	$\{c, c^{-1}\}$	$\{r, c^2r\}$	$\{cr, c^3r\}$
$\Gamma_3^{(1)}$	1	1	1	1	1
$\Gamma_3^{(2)}$	1	1	1	-1	-1
$\Gamma_3^{(3)}$	1	1	-1	1	-1
$\Gamma_3^{(4)}$	1	1	-1	-1	1
$\Gamma_3^{(5)}$	2	-2	0	0	0
ϑ_3	7	3	1	5	1

Table B. Character table for $\mathcal{G}_3$.

S1.2.4 Canonical decomposition

The completely reducible permutation representations can be decomposed into a direct sum of the irreducible representations. This is done by calculating the multiplicity m_ω of each irreducible representation (ω) using:

$$m_\omega = \frac{1}{|\mathcal{G}|} \sum_{s \in \mathcal{G}} \chi^{(\omega)}(s)^* \chi(s)$$

This leads to the following canonical decompositions:

Generations 1,2:

$$\vartheta_2 = 2\Gamma_2^{(1)} \oplus \Gamma_2^{(2)}$$

Generations 1,2,3:

$$\vartheta_3 = 3\Gamma_3^{(1)} \oplus 2\Gamma_3^{(3)} \oplus \Gamma_3^{(5)}$$

If we use $\vartheta_G^{(\omega)}$ to refer to active irreducible representations then $\vartheta_2 = 2\vartheta_2^{(1)} \oplus \vartheta_2^{(2)}$ and $\vartheta_3 = 3\vartheta_3^{(1)} \oplus 2\vartheta_3^{(2)} \oplus \vartheta_3^{(3)}$. This already suggests that there will be G active irreducible representations spanning the vector space of a tree with G generations.

S1.2.5 Symmetry-adapted basis vectors

To determine the change-of-basis matrix between the standard and natural (symmetry-adapted) variables we must project the permutation matrices onto the invariant subspaces by applying the projection operators (see p.147 [1])

$$\boldsymbol{P}^{(\omega)} = \frac{d_\omega}{|\mathcal{G}|} \sum_{s \in \mathcal{G}} \chi^{(\omega)}(s)^* \boldsymbol{D}(s)$$
$$\boldsymbol{\Pi}^{(\omega)} = \sum_{s \in \mathcal{G}} \zeta_{11}^{(\omega)}(s)^* \boldsymbol{D}(s)$$

where the first is used if $m_\omega = 1$ and the second if $m_\omega > 1$. Here $\zeta_{11}^{(\omega)}(s)$ is the first element in the irreducible representation matrix. The results are as follows:

Generations 1,2:

$$\boldsymbol{T} = \begin{pmatrix} 1 & 0 & 0 \\ 0 & \frac{1}{\sqrt{2}} & \frac{1}{\sqrt{2}} \\ 0 & \frac{1}{\sqrt{2}} & \frac{-1}{\sqrt{2}} \end{pmatrix}$$

Generations 1,2,3:

$$\boldsymbol{T} = \begin{pmatrix} 1 & 0 & 0 & 0 & 0 & 0 & 0 \\ 0 & \frac{1}{\sqrt{2}} & 0 & \frac{1}{\sqrt{2}} & 0 & 0 & 0 \\ 0 & \frac{1}{\sqrt{2}} & 0 & \frac{-1}{\sqrt{2}} & 0 & 0 & 0 \\ 0 & 0 & \frac{1}{2} & 0 & \frac{1}{2} & \frac{1}{\sqrt{2}} & 0 \\ 0 & 0 & \frac{1}{2} & 0 & \frac{1}{2} & \frac{-1}{\sqrt{2}} & 0 \\ 0 & 0 & \frac{1}{2} & 0 & \frac{-1}{2} & 0 & \frac{1}{\sqrt{2}} \\ 0 & 0 & \frac{1}{2} & 0 & \frac{-1}{2} & 0 & \frac{-1}{\sqrt{2}} \end{pmatrix}$$

S1.2.6 Covariance matrix in the symmetry-adapted basis

In the symmetry-adapted basis the covariance matrix block diagonalizes into components corresponding to each irreducible representation. The dimension of each block is the product of the multiplicity and dimensionality of that representation.

Generations 1,2:

$$\begin{aligned} \boldsymbol{\Sigma}_\Omega &= \boldsymbol{T}^{-1}\boldsymbol{\Sigma}_\mathcal{G}\boldsymbol{T} \\ &= \begin{pmatrix} \xi_{11}^{(1)} & \xi_{12}^{(1)} & \cdot \\ \xi_{12}^{(1)} & \xi_{22}^{(1)} & \cdot \\ \cdot & \cdot & \xi_{22}^{(2)} \end{pmatrix} \end{aligned}$$

Generations 1,2,3:

$$\begin{aligned} \boldsymbol{\Sigma}_\Omega &= \boldsymbol{T}^{\dagger}\boldsymbol{\Sigma}_\mathcal{G}\boldsymbol{T} \\ &= \begin{pmatrix} \xi_{11}^{(0)} & \xi_{12}^{(0)} & \xi_{13}^{(0)} & \cdot & \cdot & \cdot & \cdot \\ \xi_{12}^{(0)} & \xi_{22}^{(0)} & \xi_{23}^{(0)} & \cdot & \cdot & \cdot & \cdot \\ \xi_{13}^{(0)} & \xi_{23}^{(0)} & \xi_{33}^{(0)} & \cdot & \cdot & \cdot & \cdot \\ \cdot & \cdot & \cdot & \xi_{22}^{(1)} & \xi_{23}^{(1)} & \cdot & \cdot \\ \cdot & \cdot & \cdot & \xi_{23}^{(1)} & \xi_{33}^{(1)} & \cdot & \cdot \\ \cdot & \cdot & \cdot & \cdot & \cdot & \xi_{33}^{(2)} & 0 \\ \cdot & \cdot & \cdot & \cdot & \cdot & 0 & \xi_{33}^{(2)} \end{pmatrix} \end{aligned}$$

S1.2.7 Generalization to higher generations

For generations above $G = 3$ we can determine the symmetry group using the recursive relation:

$$\mathcal{G}_{g+1} \sim (\mathcal{G}_g \times \mathcal{G}_g) \rtimes \mathcal{S}_2,$$

The order of the group up to any generation g is thus:

$$|\mathcal{G}_{g+1}| = 2|\mathcal{G}_g|^2.$$

Given that for the base case, $|\mathcal{G}_2| = 2$, the order of the group is given explicitly by:

$$|\mathcal{G}_g| = 2^{\mathcal{A}}, \quad \mathcal{A} = 2^{(g-1)} - 1$$

Thus for pedigrees up to $g = 4$ or $g = 5$ the order of the group would be 128 and 32768, respectively. Clearly the group order increases too fast for this manual projection approach to be useful at higher generations.

However, by inspection, the following general result can be found. For a completely reducible representation on a binary tree with G generations, the number of active

irreducible representations $\mathcal{I}$ (indexed here as $0 \leq \omega \leq \mathcal{I}-1$), their dimensionalities d_ω, and multiplicities m_ω are

$$\mathcal{I} = G \tag{S5}$$

$$d_\omega = \begin{cases} 1, & \text{if } \omega = 0 \\ 2^{\omega-1}, & \text{if } \omega \geq 1 \end{cases} \tag{S6}$$

$$m_\omega = G - \omega. \tag{S7}$$

As required, this satisfies $p = \sum_{\omega=0}^{\mathcal{I}-1} m_\omega d_\omega$ when $p = 2^G - 1$.

S1.2.8 Natural variables for a tree

The natural, or symmetry-adapted, basis for the tree was interpreted in Section Generalized spectral analysis of a complete tree. A 3-integer tuple (ℓ, τ, g) was found to uniquely define each natural variable. These indices relate to the isotypic and irreducible subspaces as follows:

ℓ: **Longitudinal coordinate of source** The longitudinal coordinate of a source of variation, ℓ, corresponds to an isotypic component, ω. The fact that there are G longitudinal coordinates is consistent with there being $\mathcal{I} = G$ isotypic subspaces (Eq S5). We adopt the convention that $0 \leq \ell < G$.

τ: **Transverse coordinate of source** The transverse coordinate of a source of variation, τ, corresponds to an irreducible subspace ν within an isotypic subspace. The fact that there is 1 transverse coordinate for $\ell = 0, 1$ and $2^{\ell-1}$ coordinates for $\ell \geq 2$ (see Fig 6) is consistent with the dimensionalities d_ω found for each irreducible representation (Eq S6). We adopt the convention that $0 \leq \tau < d_\ell$.

g: **Generation** The variables within each irreducible subspace correspond to the generations g at which variation from (ℓ, τ) can be observed, where $1 \leq g \leq G$. Given that variation can only be observed in generations after the source ℓ, g is restricted to $\ell < g \leq G$ within an isotypic subspace, giving it $G - \ell$ possible values. This is consistent with the multiplicity of each irreducible representation being $m_\omega = G - \omega$ (Eq S7). Importantly, since each accessible g occurs exactly once in each irreducible subspace, the irreducible subspace consists of an ordered sequence and is thus a time series.

S1.2.9 Covariance matrix decomposition

In Eq 5, the structured covariance matrix $\boldsymbol{\Sigma}_\mathcal{G}$ was defined in terms of elements $\sigma_{gg'm}$. $\boldsymbol{\Sigma}_\Omega$ can be expressed in terms of $\sigma_{gg'm}$ by applying the similarity transform, $\boldsymbol{\Sigma}_\Omega = \boldsymbol{T}^\dagger \boldsymbol{\Sigma}_\mathcal{G} \boldsymbol{T}$. For a 3-generation tree,

$$\boldsymbol{\Sigma}_\Omega = \begin{bmatrix} \sigma_{111} & \sqrt{2}\sigma_{121} & 2\sigma_{131} & 0 & 0 & 0 & 0 \\ \sqrt{2}\sigma_{121} & \sigma_{221} + \sigma_{222} & \sqrt{2}\,(\sigma_{231} + \sigma_{232}) & 0 & 0 & 0 & 0 \\ 2\sigma_{131} & \sqrt{2}\,(\sigma_{231} + \sigma_{232}) & 2\sigma_{331} + \sigma_{332} + \sigma_{333} & 0 & 0 & 0 & 0 \\ 0 & 0 & 0 & -\sigma_{221} + \sigma_{222} & \sqrt{2}\,(-\sigma_{231} + \sigma_{232}) & 0 & 0 \\ 0 & 0 & 0 & \sqrt{2}\,(-\sigma_{231} + \sigma_{232}) & -2\sigma_{331} + \sigma_{332} + \sigma_{333} & 0 & 0 \\ 0 & 0 & 0 & 0 & 0 & -\sigma_{332} + \sigma_{333} & 0 \\ 0 & 0 & 0 & 0 & 0 & 0 & -\sigma_{332} + \sigma_{333} \end{bmatrix}$$

In Eq 18, the transformed covariance matrix $\boldsymbol{\Sigma}_\Omega$ was defined in terms of elements $\xi_{gg'}^{(\ell)}$. $\boldsymbol{\Sigma}_\mathcal{G}$ can be expressed in terms of $\xi_{gg'}^{(\ell)}$ by applying the reverse similarity transform, $\boldsymbol{\Sigma}_\mathcal{G} = \boldsymbol{T} \boldsymbol{\Sigma}_\Omega \boldsymbol{T}^\dagger$. For a 3-generation tree,

$$
\boldsymbol{\Sigma}_{\mathcal{G}} = \begin{bmatrix}
\xi_{11}^{(0)} & \frac{\sqrt{2}\xi_{12}^{(0)}}{2} & \frac{\sqrt{2}\xi_{12}^{(0)}}{2} & \frac{\xi_{13}^{(0)}}{2} & \frac{\xi_{13}^{(0)}}{2} & \frac{\xi_{13}^{(0)}}{2} & \frac{\xi_{13}^{(0)}}{2} \\
\frac{\sqrt{2}\xi_{12}^{(0)}}{2} & \frac{\xi_{22}^{(0)}}{2} + \frac{\xi_{22}^{(1)}}{2} & \frac{\xi_{22}^{(0)}}{2} - \frac{\xi_{22}^{(1)}}{2} & \frac{\sqrt{2}\xi_{23}^{(0)}}{4} + \frac{\sqrt{2}\xi_{23}^{(1)}}{4} & \frac{\sqrt{2}\xi_{23}^{(0)}}{4} + \frac{\sqrt{2}\xi_{23}^{(1)}}{4} & \frac{\sqrt{2}\xi_{23}^{(0)}}{4} - \frac{\sqrt{2}\xi_{23}^{(1)}}{4} & \frac{\sqrt{2}\xi_{23}^{(0)}}{4} - \frac{\sqrt{2}\xi_{23}^{(1)}}{4} \\
\frac{\sqrt{2}\xi_{12}^{(0)}}{2} & \frac{\xi_{22}^{(0)}}{2} - \frac{\xi_{22}^{(1)}}{2} & \frac{\xi_{22}^{(0)}}{2} + \frac{\xi_{22}^{(1)}}{2} & \frac{\sqrt{2}\xi_{23}^{(0)}}{4} - \frac{\sqrt{2}\xi_{23}^{(1)}}{4} & \frac{\sqrt{2}\xi_{23}^{(0)}}{4} - \frac{\sqrt{2}\xi_{23}^{(1)}}{4} & \frac{\sqrt{2}\xi_{23}^{(0)}}{4} + \frac{\sqrt{2}\xi_{23}^{(1)}}{4} & \frac{\sqrt{2}\xi_{23}^{(0)}}{4} + \frac{\sqrt{2}\xi_{23}^{(1)}}{4} \\
\frac{\xi_{13}^{(0)}}{2} & \frac{\sqrt{2}\xi_{23}^{(0)}}{4} + \frac{\sqrt{2}\xi_{23}^{(1)}}{4} & \frac{\sqrt{2}\xi_{23}^{(0)}}{4} - \frac{\sqrt{2}\xi_{23}^{(1)}}{4} & \frac{\xi_{33}^{(0)}}{4} + \frac{\xi_{33}^{(1)}}{4} + \frac{\xi_{33}^{(2)}}{2} & \frac{\xi_{33}^{(0)}}{4} + \frac{\xi_{33}^{(1)}}{4} - \frac{\xi_{33}^{(2)}}{2} & \frac{\xi_{33}^{(0)}}{4} - \frac{\xi_{33}^{(1)}}{4} & \frac{\xi_{33}^{(0)}}{4} - \frac{\xi_{33}^{(1)}}{4} \\
\frac{\xi_{13}^{(0)}}{2} & \frac{\sqrt{2}\xi_{23}^{(0)}}{4} + \frac{\sqrt{2}\xi_{23}^{(1)}}{4} & \frac{\sqrt{2}\xi_{23}^{(0)}}{4} - \frac{\sqrt{2}\xi_{23}^{(1)}}{4} & \frac{\xi_{33}^{(0)}}{4} + \frac{\xi_{33}^{(1)}}{4} - \frac{\xi_{33}^{(2)}}{2} & \frac{\xi_{33}^{(0)}}{4} + \frac{\xi_{33}^{(1)}}{4} + \frac{\xi_{33}^{(2)}}{2} & \frac{\xi_{33}^{(0)}}{4} - \frac{\xi_{33}^{(1)}}{4} & \frac{\xi_{33}^{(0)}}{4} - \frac{\xi_{33}^{(1)}}{4} \\
\frac{\xi_{13}^{(0)}}{2} & \frac{\sqrt{2}\xi_{23}^{(0)}}{4} - \frac{\sqrt{2}\xi_{23}^{(1)}}{4} & \frac{\sqrt{2}\xi_{23}^{(0)}}{4} + \frac{\sqrt{2}\xi_{23}^{(1)}}{4} & \frac{\xi_{33}^{(0)}}{4} - \frac{\xi_{33}^{(1)}}{4} & \frac{\xi_{33}^{(0)}}{4} - \frac{\xi_{33}^{(1)}}{4} & \frac{\xi_{33}^{(0)}}{4} + \frac{\xi_{33}^{(1)}}{4} + \frac{\xi_{33}^{(2)}}{2} & \frac{\xi_{33}^{(0)}}{4} + \frac{\xi_{33}^{(1)}}{4} - \frac{\xi_{33}^{(2)}}{2} \\
\frac{\xi_{13}^{(0)}}{2} & \frac{\sqrt{2}\xi_{23}^{(0)}}{4} - \frac{\sqrt{2}\xi_{23}^{(1)}}{4} & \frac{\sqrt{2}\xi_{23}^{(0)}}{4} + \frac{\sqrt{2}\xi_{23}^{(1)}}{4} & \frac{\xi_{33}^{(0)}}{4} - \frac{\xi_{33}^{(1)}}{4} & \frac{\xi_{33}^{(0)}}{4} - \frac{\xi_{33}^{(1)}}{4} & \frac{\xi_{33}^{(0)}}{4} + \frac{\xi_{33}^{(1)}}{4} - \frac{\xi_{33}^{(2)}}{2} & \frac{\xi_{33}^{(0)}}{4} + \frac{\xi_{33}^{(1)}}{4} + \frac{\xi_{33}^{(2)}}{2}
\end{bmatrix}
$$

Elements along the diagonal represent the components of variance referred to in Eq 31. Boxes indicate the covariance matrices for generation 2 only (dotted) and generation 3 only (dashed).

S1.3 Sparsity

Having employed the symmetry constraint in Section "Complexity of the structured covariance", we remarked that the number of replicates required, n_{min}, still grew with the number of generations. To identify another constraint, we note that the $G - \ell$ natural variables in each irreducible subspace (ℓ, τ) represent a time series from generation $\ell + 1$ to G (see Section "Generalized spectral analysis of a complete tree"). Together, the unique irreducible subspaces comprise a set of G independent time series each starting at a different generation but all ending at G. The additional structure we impose is to consider each of these time series as a fixed order Markov chain.

We note first that the structure of $\boldsymbol{\Sigma}_{\mathcal{G}}$ is preserved in its inverse, $\boldsymbol{K}_{\mathcal{G}} = \boldsymbol{\Sigma}_{\mathcal{G}}^{-1}$, a consequence of $\mathcal{G}$-invariance [3]. This means that the spectral precision matrix, $\boldsymbol{K}_{\Omega} = \boldsymbol{T}^{\dagger}\boldsymbol{K}_{\mathcal{G}}\boldsymbol{T}$ has the same block-diagonal structure as $\boldsymbol{\Sigma}_{\Omega}$. Hence each irreducible block $\boldsymbol{K}_{\Omega}^{(\ell)}$ in the spectral precision matrix is just the inverse of the corresponding irreducible block in the spectral covariance $\boldsymbol{\Sigma}_{\Omega}^{(\ell)}$:

$$\boldsymbol{K}_{\Omega}^{(\ell)} = \left[\boldsymbol{\Sigma}_{\Omega}^{(\ell)}\right]^{-1}. \tag{S8}$$

Imposing a Markov constraint on $\boldsymbol{\Sigma}_{\Omega}^{(\ell)}$ involves imposing a sparsity constraint on $\boldsymbol{K}_{\Omega}^{(\ell)}$. More specifically, matrix elements in $\boldsymbol{K}_{\Omega}^{(\ell)}$ outside a diagonal band (the tri-diagonal in the case of a 1st order Markov process) are constrained to be zero. Remember that it is the structure of each $\boldsymbol{K}_{\Omega}^{(\ell)}$ that is sparse; the precision matrix itself, $\boldsymbol{K}_{\mathcal{G}}$, may not be particularly sparse. We remark that a zero in the precision matrix enforces conditional uncorrelatedness between two variables without assuming Gaussianity (if the distribution is Gaussian, then this pair of variables is also conditionally independent).

A restricted-order Markov chain is a simple case of a decomposable graphical model [4, 5] and thus yields an explicit estimate of the covariance matrix. Following the procedure for a decomposable model, we organize variables in the irreducible block into cliques and separators, a straightforward exercise for a Markov chain of any order. If $\boldsymbol{S}_{\Omega}^{(\ell)}$ represents the irreducible block of the sample covariance, we can label sub-blocks of cliques and separators within $\boldsymbol{S}_{\Omega}^{(\ell)}$ as

$$\boldsymbol{S}_{\Omega,c_i}^{(\ell)},\ i = 1, ..., \mathcal{N}_C; \quad \boldsymbol{S}_{\Omega,s_i}^{(\ell)},\ i = 2, ..., \mathcal{N}_C$$

where the subscript c_i refers to a clique, s_i refers to a separator, and $\mathcal{N}_C$ is the number of cliques in the irreducible block. The covariance estimate for an irreducible block is

then given by (p.145 [5])

$$\hat{\boldsymbol{K}}_{\Omega}^{(\ell)} = \sum_{i=1}^{\mathcal{N}_C} \left\{ \left[\boldsymbol{S}_{\Omega,c_i}^{(\ell)} \right]^{-1} \right\}^0 - \sum_{i=2}^{\mathcal{N}_C} \left\{ \left[\boldsymbol{S}_{\Omega,s_i}^{(\ell)} \right]^{-1} \right\}^0 \tag{S9}$$

$$\hat{\boldsymbol{\Sigma}}_{\Omega}^{(\ell)} = \left[\hat{\boldsymbol{K}}_{\Omega}^{(\ell)} \right]^{-1} \tag{S10}$$

where the expression $\{\Upsilon\}^0$ denotes a matrix with the dimensions of $\hat{\boldsymbol{K}}_{\Omega}^{(\ell)}$ which has its appropriate sub-block occupied by Υ and zeros elsewhere. Recombining the irreducible blocks using a direct sum gives the Markov-constrained spectral covariance estimate, $\hat{\boldsymbol{\Sigma}}_{\Omega}$:

$$\begin{aligned} \hat{\boldsymbol{\Sigma}}_{\Omega} &= \bigoplus_{\ell=0}^{G-1} \left[\bigoplus_{\tau=0}^{d_\ell - 1} \hat{\boldsymbol{\Sigma}}_{\Omega}^{(\ell)} \right], \\ &= \bigoplus_{\ell=0}^{G-1} \left[I_{d_\ell} \otimes \hat{\boldsymbol{\Sigma}}_{\Omega}^{(\ell)} \right], \quad d_\ell = \begin{cases} 1, & \text{if } \ell = 0 \\ 2^{\ell-1}, & \text{if } \ell \geq 1 \end{cases}, \end{aligned} \tag{S11}$$

from which the Markov-constrained structured covariance, $\hat{\boldsymbol{\Sigma}}_{\mathcal{G}}$, can be calculated using the inverse transform:

$$\hat{\boldsymbol{\Sigma}}_{\mathcal{G}} = \boldsymbol{T} \hat{\boldsymbol{\Sigma}}_{\Omega} \boldsymbol{T}^{\dagger}. \tag{S12}$$

Eq S9 shows that, since it is the inverse of the clique and separator sub-blocks that are required (rather than the entire irreducible block), it is only these sub-blocks (with maximum dimension $\mathcal{M} + 1$) that need to be positive definite. The minimum number of replicates required for positive definiteness is thus set by the order $\mathcal{M}$ of the Markov process, which is fixed, rather than by the size of the irreducible block, which grows linearly with G. In general then, $n_{\min} = \mathcal{M} + 2$ and we have finally achieved our goal of having the data requirements be independent of the number of generations being analyzed. Note that restricting the non-zero parameters in the precision matrix to be on the diagonal band means that $\mathcal{N}_\Sigma \sim \mathcal{O}(G^2)$, down from the cubic dependence in Eq 20. p_{eff} remains unchanged (Eq 19).

Inspection of the T-cell and worm lineage data show that, at least up to generation 4, non-zero values in $\boldsymbol{K}_{\Omega}^{(\ell)}$ are indeed primarily confined to the tri-diagonal, justifying the (first-order) Markov process assumption.

S1.4 Missing data

The EM algorithm iteratively improves the estimate of the covariance matrix, generating expected values of the sufficient statistics at each step.

In more detail (p.223 [6]), the first and second order statistics are calculated for each replicate i by partitioning the variables into observed sets, labelled o_i, and unobserved sets, labelled u_i. Members of each set usually differ from one replicate to the next. The vector of unobserved values in each replicate is then filled by its expected value conditioned on the vector of observed values:

$$\begin{aligned} \boldsymbol{Y}_{i,u_i} &= \mathbb{E}(\boldsymbol{Y}_{i,u_i} | \boldsymbol{Y}_{i,o_i}) \\ &= \hat{\boldsymbol{\mu}}_{u_i} + \hat{\boldsymbol{\Sigma}}_{u_i,o_i} \hat{\boldsymbol{\Sigma}}_{o_i,o_i}^{-1} \left(\boldsymbol{Y}_{i,o_i} - \hat{\boldsymbol{\mu}}_{o_i} \right). \end{aligned} \tag{S13}$$

Combining these with the observed values completes the first order statistic, $\boldsymbol{Y}_i = \{\boldsymbol{Y}_{i,o_i}, \boldsymbol{Y}_{i,u_i}\}$ for i.

The second order statistic $(\boldsymbol{YY})_i$ for each replicate i, partitioned into observed and unobserved sections, is found from

$$\begin{aligned}
(\boldsymbol{YY}')_{i,o_io_i} &= \boldsymbol{Y}_{i,o_i}\boldsymbol{Y}'_{i,o_i} \\
(\boldsymbol{YY}')_{i,u_io_i} &= \boldsymbol{Y}_{i,u_i}\boldsymbol{Y}'_{i,o_i} \\
(\boldsymbol{YY}')_{i,o_iu_i} &= \boldsymbol{Y}_{i,o_i}\boldsymbol{Y}'_{i,u_i} \\
(\boldsymbol{YY}')_{i,u_iu_i} &= \boldsymbol{Y}_{i,u_i}\boldsymbol{Y}'_{i,u_i} + \hat{\boldsymbol{\Sigma}}_{u_iu_i|o_io_i},
\end{aligned} \tag{S14}$$

where

$$\hat{\boldsymbol{\Sigma}}_{u_iu_i|o_io_i} = \hat{\boldsymbol{\Sigma}}_{u_i,u_i} - \hat{\boldsymbol{\Sigma}}_{u_i,o_i}\hat{\boldsymbol{\Sigma}}^{-1}_{o_i,o_i}\hat{\boldsymbol{\Sigma}}_{o_i,u_i}$$

is the residual covariance of the unobserved variables after conditioning on the observed variables.

Once this exercise has been completed for all replicates, the sample mean and covariance are calculated from the usual

$$\overline{\boldsymbol{y}} = \frac{1}{n}\sum_{i=1}^{n}\boldsymbol{Y}_i, \quad \boldsymbol{S} = \frac{1}{n}\sum_{i=1}^{n}(\boldsymbol{YY}')_i - \hat{\boldsymbol{\mu}}\hat{\boldsymbol{\mu}}'. \tag{S15}$$

Finally, symmetry constraints are applied to $\overline{\boldsymbol{y}}$ to determine $\hat{\boldsymbol{\mu}}_{\mathcal{G}}$ while symmetry and sparsity constraints are applied to $\boldsymbol{S}$ to determine and $\hat{\boldsymbol{\Sigma}}_{\mathcal{G}}$ (see Section "Summary of algorithm").

S1.5 Maximum likelihood estimation

The second order theory given in Section "Covariance estimation" for complete data is a nonparametric spectral estimate of $\boldsymbol{\Sigma}$. It does not assume a probability distribution. Here we show that our estimates $\hat{\boldsymbol{\Sigma}}_{\mathcal{G}}$ and $\hat{\boldsymbol{\Sigma}}_{\Omega}$ are in fact the maximum likelihood estimates for a multivariate Gaussian. This becomes important when we have to assume a distribution to account for missing data.

In this study the traits we examine for T cells and *C. elegans* are continuous and approximately marginally Gaussian. Thus it is reasonable to assume that the joint probability distribution over the complete tree $\mathcal{P}(\boldsymbol{y})$, where $\boldsymbol{y}$ is a p-dimensional random variable representing the single trait for each lineal position, can be modeled by the multivariate Gaussian

$$\mathcal{P}(\boldsymbol{y};\boldsymbol{\mu},\boldsymbol{\Sigma}) = \frac{|\boldsymbol{\Sigma}|^{-1/2}}{(2\pi)^{p/2}}\exp\left[-\frac{1}{2}(\boldsymbol{y}-\boldsymbol{\mu})'\boldsymbol{\Sigma}^{-1}(\boldsymbol{y}-\boldsymbol{\mu})\right],$$

where $\boldsymbol{\Sigma}$ is the variance-covariance matrix and $\boldsymbol{\mu}$ is the multivariate mean.

The sample mean ($\overline{\boldsymbol{y}}$) and (biased) sample covariance ($\boldsymbol{S}$) are given by

$$\overline{\boldsymbol{y}} = \frac{1}{n}\sum_{i=1}^{n}\boldsymbol{Y}_i, \quad \boldsymbol{S} = \frac{1}{n}\sum_{i=1}^{n}\boldsymbol{Y}_i\boldsymbol{Y}_i' - \overline{\boldsymbol{y}}\,\overline{\boldsymbol{y}}', \tag{S16}$$

where $\boldsymbol{Y}_i$ is the data vector from pedigree i. As usual, the unstructured mean and variance-covariance maximum likelihood estimates (MLE) are given simply by $\hat{\boldsymbol{\mu}} = \overline{\boldsymbol{y}}$ and $\hat{\boldsymbol{\Sigma}} = \boldsymbol{S}$.

We now find the MLE for a covariance matrix constrained to have the structure of an unordered tree (e.g. Eq 5). Crucially, since the structure of $\boldsymbol{\Sigma}$ arises from a group symmetry, its inverse $\boldsymbol{K} = \boldsymbol{\Sigma}^{-1}$ has the same structure [3]. With the constraint being

in $\boldsymbol{K}$, the MLE has an explicit form [7,8]. Consider the log-likelihood of the multivariate Gaussian

$$\begin{aligned}\mathcal{L}(\boldsymbol{\mu}, \boldsymbol{K}; \boldsymbol{Y}) &= \ln \mathcal{P}(\boldsymbol{Y}; \boldsymbol{\mu}, \boldsymbol{K}) \\ &= \frac{n}{2}\left[\ln\det \boldsymbol{K} - \operatorname{tr}\left(\boldsymbol{S}\boldsymbol{K}\right) - p\ln(2\pi)\right]\end{aligned} \tag{S17}$$

over positive definite matrices $\boldsymbol{K}$. To constrain the structure of $\boldsymbol{K}$ we represent it as a linear combination of matrices. The resulting structured inverse covariance is then

$$\boldsymbol{K}_{\mathcal{G}} = \sum_{\alpha} a_{\alpha}\boldsymbol{A}_{\alpha}, \tag{S18}$$

where each $\boldsymbol{A}_{\alpha}$ is a matrix of 0's and 1's which has the same dimensions as $\boldsymbol{K}_{\mathcal{G}}$, with 1's identifying a particular shared parameter and a_{α} giving the value of that parameter.

The MLE is found by differentiating Eq S17 with respect to each a_{α} and setting $d\mathcal{L}/da_{\alpha} = 0$, giving

$$\frac{d}{da_{\alpha}}\ln\det \boldsymbol{K}_{\mathcal{G}} = \frac{d}{da_{\alpha}}\operatorname{tr}(\boldsymbol{S}\boldsymbol{K}_{\mathcal{G}}). \tag{S19}$$

Substituting Eq S18 gives

$$\operatorname{tr}(\hat{\boldsymbol{\Sigma}}_{\mathcal{G}}\boldsymbol{A}_{\alpha}) = \operatorname{tr}\left(\boldsymbol{S}\boldsymbol{A}_{\alpha}\right). \tag{S20}$$

Since the matrices are symmetric we can equate the inner products of the matrices:

$$\langle \hat{\boldsymbol{\Sigma}}_{\mathcal{G}}, \boldsymbol{A}_{\alpha}\rangle = \langle \boldsymbol{S}, \boldsymbol{A}_{\alpha}\rangle. \tag{S21}$$

Thus the MLE of each shared parameter in $\hat{\boldsymbol{\Sigma}}_{\mathcal{G}}$ is found by averaging the corresponding elements in $\boldsymbol{S}$ [7]. The result, $\boldsymbol{S}_{\mathcal{G}}$, is thus the MLE of the structured covariance

$$\hat{\boldsymbol{\Sigma}}_{\mathcal{G}} = \boldsymbol{S}_{\mathcal{G}}. \tag{S22}$$

Similarly, for a multivariate Gaussian with group symmetries the MLE of the mean, $\hat{\boldsymbol{\mu}}$, is given explicitly [9]. For the case of a binary tree $\hat{\boldsymbol{\mu}}$ is found by pooling data from lineal positions in the same generation.

S1.5.1 MLE from irreducible components

The decomposition of $\boldsymbol{\Sigma}_{\mathcal{G}}$ into block-diagonal form reduces the single MLE calculation over all p variables into several smaller independent MLE calculations. To see this, let $\boldsymbol{K} \in \mathcal{W}_{\mathcal{G}}$. Then from Eq S3, $\boldsymbol{K}$ can be decomposed into irreducible blocks $K_{\Omega}^{(\ell)}$. The parts of the likelihood function thus become

$$\begin{aligned}\ln\det \boldsymbol{K} = \ln\det \boldsymbol{T}^{\dagger}\boldsymbol{K}\boldsymbol{T} &= \ln\det \boldsymbol{K}_{\Omega}, \\ &= \sum_{\ell=0}^{G-1} d_{\ell}\ln\det K_{\Omega}^{(\ell)},\end{aligned} \tag{S23}$$

and

$$\begin{aligned}\operatorname{tr}\left(\boldsymbol{S}\boldsymbol{K}\right) = \operatorname{tr}\left(\boldsymbol{T}^{\dagger}\boldsymbol{S}\boldsymbol{T}\boldsymbol{T}^{\dagger}\boldsymbol{K}\boldsymbol{T}\right) &= \operatorname{tr}\left(\boldsymbol{S}_{\Omega}\boldsymbol{K}_{\Omega}\right), \\ &= \sum_{\ell=0}^{G-1}\sum_{\tau=0}^{d_{\ell}-1}\langle S_{\Omega}^{(\ell,\tau)}, K_{\Omega}^{(\ell)}\rangle,\end{aligned} \tag{S24}$$

where $K_\Omega^{(\ell)}$ has the same dimensionality as $S_\Omega^{(\ell,\tau)}$ but is independent of τ. Substituting Eqs. S23 and S24 into the likelihood Eq S17, it is thus apparent that each irreducible block can be treated as an independent MLE calculation. The result,

$$\hat{\mathbf{\Sigma}}_\Omega^{(\ell)} = \frac{1}{d_\ell} \sum_{\tau=0}^{d_\ell - 1} S_\Omega^{(\ell,\tau)} = \overline{S_\Omega^{(\ell)}} \tag{S25}$$

states that $\hat{\mathbf{\Sigma}}_\Omega^{(\ell)}$ is found by averaging the d_ℓ irreducible subblocks in the sample spectral covariance. This procedure ensures that elements of $\boldsymbol{S}_\Omega$ that are outside the block diagonal are ignored.

The resulting $\hat{\mathbf{\Sigma}}$ can be reconstructed by substituting Eq S25 in Eq S3 and transforming back to the original basis:

$$\hat{\mathbf{\Sigma}}_\Omega = \bigoplus_{\ell=0}^{G-1} \left[\bigoplus_{\tau=0}^{d_\ell - 1} \hat{\mathbf{\Sigma}}_\Omega^{(\ell)} \right] = \bigoplus_{\ell=0}^{G-1} \left[I_{d_\ell} \otimes \hat{\mathbf{\Sigma}}_\Omega^{(\ell)} \right], \tag{S26}$$

$$\hat{\mathbf{\Sigma}} = \boldsymbol{T} \hat{\mathbf{\Sigma}}_\Omega \boldsymbol{T}^\dagger \tag{S27}$$

The procedure for finding $\hat{\mathbf{\Sigma}}$ for a $\mathcal{G}$-invariant covariance is thus as follows:

1. Transform $\boldsymbol{S}$ into the symmetry-adapted basis.
2. Zero the elements outside the irreducible blocks.
3. If there is more than one irreducible block per isotypic block, average them.
4. Transform back to the original basis.

Importantly, Eq S25 gives an explicit MLE of the irreducible block that involves simply re-arranging terms in the sufficient statistic $\boldsymbol{S}$. Each of these blocks is thus a descriptive statistic for tree-structured data, involving linear combinations of data points, sums of squares and no parameters. Thus the MLE is the same as our original nonparametric estimate.

S1.6 Lineage nomenclature for *C. elegans*

The spectral features found in Fig 11b can be related to known subtrees in the *C. elegans* lineage. Fig A shows the *PHA-4* gene expression pattern [10] on a lineage where each lineal position has been given its standard label [11]. Red dashed lines are used to highlight a few of the subtrees giving rise to particularly strong bifurcated expression in generation 8.

References

1. Stiefel E, Fässler A. Group Theoretical Methods and Their Applications. Birkhäuser Boston; 1992. Available from: https://books.google.com.au/books?id=bMKBllvEDTkC.
2. Shah P, Chandrasekaran V. Group symmetry and covariance regularization. Electron J Statist. 2012;6:1600–1640.
3. Proof: If $\mathbf{\Sigma} \in \mathcal{W}_\mathcal{G}$ then $\boldsymbol{D}_s \mathbf{\Sigma} \boldsymbol{D}_s' = \mathbf{\Sigma}, \forall s \in \mathcal{G}$ (Eq. 6). Since $\boldsymbol{D}_s^{-1} = \boldsymbol{D}_s'$, it follows that $\boldsymbol{D}_s \mathbf{\Sigma}^{-1} \boldsymbol{D}_s' = \mathbf{\Sigma}^{-1}, \forall s \in \mathcal{G}$ and hence $\mathbf{\Sigma}^{-1} \in \mathcal{W}_\mathcal{G}$;.

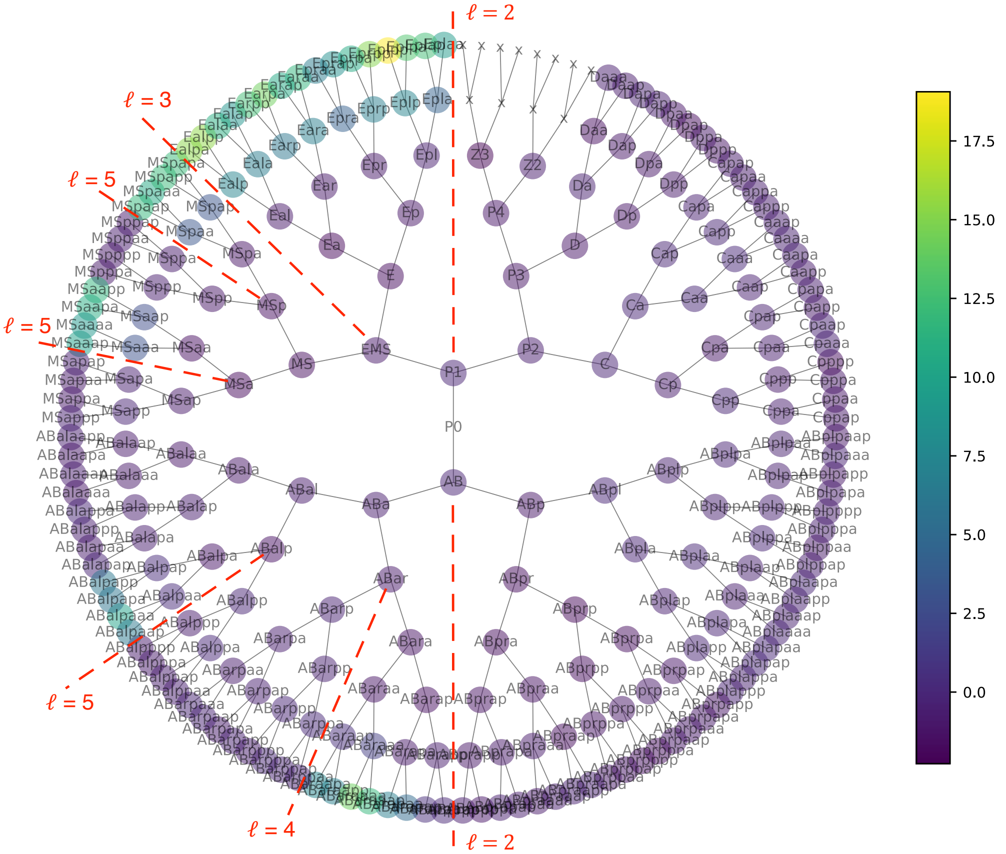

Figure A. Strength of expression of the *PHA-4* gene, a marker for pharyngeal and intestinal tissue, in the first 8 generations of embryogenesis in *C. elegans*. Each lineal position is labelled with its standard identifier [11]. Red dashed lines highlight particular subtrees ℓ that give rise to bifurcated expression at generation 8. For example, the division of P1 (into EMS and P2) occurs at $\ell = 2$. Since there is no *PHA-4* expression (at generation 8) among the descendants of P2 and significant *PHA-4* expression in the descendants of EMS, strong bifurcated expression is detected at $\ell = 2$, as illustrated by the peak in Fig 11b. This $\ell = 2$ peak is further enhanced by fate bifurcation upon division of AB, where descendants of ABa exhibit pharyngeal fate while the descendants of its sister, ABp, do not. Division of EMS into E (all of whose descendants are intestinal cells) and MS (some of whose descendants are pharyngeal cells) provides a further signal at $\ell = 3$. Heterogeneity at $\ell = 5$ arises in sublineages of E, MS, and ABa, some of which are highlighted. Though this pattern is essentially invariant, its non-clonal structure means that we should attribute fate to multiple lineal positions.

4. Speed TP, Kiiveri HT. Gaussian Markov Distributions over Finite Graphs. Ann Statist. 1986;14(1):138–150.

5. Lauritzen SL. Graphical Models. Oxford Statistical Science Series. Clarendon Press; 1996. Available from: https://books.google.com.au/books?id=mGQWkx4guhAC.

6. Little RJA, Rubin DB. Statistical Analysis with Missing Data. Wiley Series in Probability and Statistics. Wiley; 2002. Available from: https://books.google.com.au/books?id=aYPwAAAAMAAJ.

7. Hojsgaard S, Lauritzen SL. Graphical Gaussian models with edge and vertex symmetries. Journal of the Royal Statistical Society: Series B (Statistical Methodology). 2008;70(5):1005–1027.

8. Szatrowski TH. Necessary and Sufficient Conditions for Explicit Solutions in the Multivariate Normal Estimation Problem for Patterned Means and Covariances. The Annals of Statistics. 1980;8(4):802–810.

9. Gehrmann H, Lauritzen SL. Estimation of means in graphical Gaussian models with symmetries. Ann Statist. 2012;40(2):1061–1073. doi:10.1214/12-AOS991.

10. Santella A, Kovacevic I, Herndon LA, Hall DH, Du Z, Bao Z. Digital development: a database of cell lineage differentiation in C. elegans with lineage phenotypes, cell-specific gene functions and a multiscale model. Nucleic Acids Research. 2016;44(D1):D781–D785. doi:10.1093/nar/gkv1119.

11. Sulston JE, Schierenberg E, White JG, Thomson JN. The embryonic cell lineage of the nematode Caenorhabditis elegans. Developmental Biology. 1983;100(1):64 – 119.
